# 1-Aminocyclopropane-1-Carboxylic Acid Oxidase (ACO): The Enzyme That Makes the Plant Hormone Ethylene

**DOI:** 10.3389/fpls.2019.00695

**Published:** 2019-05-29

**Authors:** Maarten Houben, Bram Van de Poel

**Affiliations:** Molecular Plant Hormone Physiology Laboratory, Division of Crop Biotechnics, Department of Biosystems, KU Leuven, Leuven, Belgium

**Keywords:** ethylene biosynthesis, 1-aminocyclopropane-1-carboxylate oxidase, transcriptional and post-translation regulation, phylogeny, physiology

## Abstract

The volatile plant hormone ethylene regulates many plant developmental processes and stress responses. It is therefore crucial that plants can precisely control their ethylene production levels in space and time. The ethylene biosynthesis pathway consists of two dedicated steps. In a first reaction, S-adenosyl-L-methionine (SAM) is converted into 1-aminocyclopropane-1-carboxylic acid (ACC) by ACC-synthase (ACS). In a second reaction, ACC is converted into ethylene by ACC-oxidase (ACO). Initially, it was postulated that ACS is the rate-limiting enzyme of this pathway, directing many studies to unravel the regulation of ACS protein activity, and stability. However, an increasing amount of evidence has been gathered over the years, which shows that ACO is the rate-limiting step in ethylene production during certain dedicated processes. This implies that also the ACO protein family is subjected to a stringent regulation. In this review, we give an overview about the state-of-the-art regarding ACO evolution, functionality and regulation, with an emphasis on the transcriptional, post-transcriptional, and post-translational control. We also highlight the importance of ACO being a prime target for genetic engineering and precision breeding, in order to control plant ethylene production levels.

## Introduction on Ethylene Biosynthesis

Ethylene was the first gaseous hormone to be discovered in plants. It is an important regulator of many developmental and physiological processes such as seed dormancy, germination, vegetative growth, flowering, climacteric fruit ripening, and senescence. Additionally, ethylene was shown to play an important role in the plant’s defense against biotic and abiotic stress factors (Lin et al., 2009; Van de Poel et al., 2015; Wen, 2015).

**FIGURE 1:**
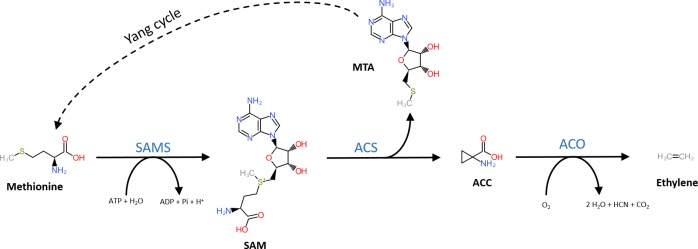
Structural representation of the ethylene biosynthesis pathway. Methionine is converted into SAM by SAM synthetase (SAMS) requiring ATP. Next, SAM is converted into methylthioadenosine (MTA) and 1-aminocyclopropane-1-carboxylic acid (ACC) by ACC-synthase (ACS). MTA is recycled back to methionine by the Yang-cycle (several reactions depicted by the dotted line). In the final step, ACC-oxidase (ACO) catalyzes the release of ethylene from ACC using molecular oxygen.

The general precursor of the ethylene biosynthesis pathway is the amino acid methionine (Figure 1; Lieberman et al., 1966). In a first, but general reaction, methionine is converted into S-adenosyl-L-methionine (SAM) by SAM synthetase using ATP (Adams and Yang, 1977). The subsequent reaction steps are unique to the ethylene biosynthesis pathway. First, SAM is converted into 1-aminocyclopropane-1-carboxylic acid (ACC) and 5′-methylthioadenosine (MTA) by ACC-synthase (ACS) (Murr and Yang, 1975; Adams and Yang, 1979; Boller et al., 1979). ACS is a member of the pyridoxal-5′-phosphate (PLP) dependent aminotransferases, which use PLP as a co-factor (Boller et al., 1979). The side product MTA is recycled back to methionine by the Yang cycle to avoid a depletion of methionine during high rates of ethylene production (Murr and Yang, 1975). More details on the different steps of the Yang cycle are presented in Bürstenbinder et al. (2010) and Pommerrenig et al. (2011). In a second step, ethylene is released from ACC by ACC-oxidase (ACO) (Hamilton et al., 1990; Ververidis and John, 1991), a reaction that requires molecular oxygen (Burg and Burg, 1965). Alternatively, ACC can be converted to malonyl-ACC (MACC; Amrhein et al., 1981), γ-glutamyl-ACC (GACC; Martin and Saftner, 1995), and jasmonyl-ACC (JA-ACC; Staswick and Tiryaki, 2004). An in-depth review on the derivatization of ACC is given by Van de Poel and Van Der Straeten (2014).

## The Discovery of ACO and Its Reaction Mechanism

For a long time it remained extremely difficult to purify ACO (formerly named the Ethylene Forming Enzyme, EFE) and determine its *in vitro* activity, mainly because it was thought that ACO was a membrane bound protein that lost its activity upon homogenization (Kende, 1989). Some residual or partial *in vitro* ACO activity was retained in membrane preparations of pea (Guy and Kende, 1984; Porter et al., 1986), bean (Guy and Kende, 1984; Mayne and Kende, 1986), Sprenger’s asparagus (Porter et al., 1986) and kiwi fruit (Mitchell et al., 1988), which was only a fraction (5–0.5%) of the total *in vivo* ethylene production capacity. A breakthrough was made when the clone pTOM13 was characterized to code for a putative *ACO* gene of tomato (Hamilton et al., 1990). The elucidation of the protein sequence of this first ACO allowed Ververidis and John to find sequence similarity with a flavonone 3-hydroxylase of snapdragon (*Antirrhinum majus*). This homology made them realize that both iron and ascorbic acid could be essential for ACO enzyme activity. This insight made Ververidis and John (1991) the first to successfully extract and quantify *in vitro* ACO activity from melon fruit tissue.

Iron, in the form of Fe(II), is an essential metal cofactor, which is required for ACO enzyme activity (Bouzayen et al., 1991). Iron participates by coordinating the binding of the amino group of ACC to H177 and the carboxylate group of ACC to D179, which are two critical ACO residues in the reaction center (Zhang et al., 2004; Tierney et al., 2005; Brisson et al., 2012). The ascorbate cofactor is used as a reductant to catalyze the opening of the ACC-ring (Zhang et al., 2004; Murphy et al., 2014). The ACO reaction mechanism also uses molecular oxygen and bicarbonate as activators in order to catalyze the conversion of ACC into ethylene (Adams and Yang, 1981; Peiser et al., 1984). During this reaction, an unstable intermediate cyanoformate ion [(NCCO_2_)^-^] is formed, which rapidly decomposes in CO_2_ and CN^-^ (Murphy et al., 2014). The reactive cyanide ion (CN^-^) is subsequently detoxified into β-cyanoalanine (Peiser et al., 1984; Dilley et al., 2013; Murphy et al., 2014).

ACC-oxidase is a member of the 2-oxoglutarate-dependent dioxygenase (2OGD) superfamily of non-heme iron-containing proteins (Kawai et al., 2014). The 2OGD superfamily is one of the largest enzyme families in plants, with most of its members being active in oxygenation and hydroxylation reactions (Kawai et al., 2014). Nonetheless, 2OGD enzymes can have more diverse roles and participate for example in demethylations, desaturations, ring closure, ring cleavage, epimerization, rearrangement, halogenation, and demethylenation reactions in plants (Farrow and Facchini, 2014). Characteristic for all 2OGDs is the double-stranded β-helix (DSBH) core fold, which contains a typical 2-His-1-carboxylate motif required for iron binding, also encountered in ACO. This motif consists of two His residues and the carboxylate group from an Asp or a Glu residue, and is responsible for the ligation of Fe(II) in the enzyme catalytic site, and thus critical for ACC binding (Aik et al., 2015; Martinez and Hausinger, 2015; Murphy et al., 2014).

Despite the fact that 2OGD enzymes are typically localized in the cytosol (Kawai et al., 2014), the exact subcellular localization of ACO remains a matter of debate. Some studies have suggested that ACO is localized at the plasma membrane (Rombaldi et al., 1994; Ramassamy et al., 1998), as originally postulated (Kende, 1989). However, other studies have shown that ACO is localized in the cytosol (Peck et al., 1992; Reinhardt et al., 1994; Chung et al., 2002; Hudgins et al., 2006), matching the general localization of 2OGD enzymes. Other studies have measured ACO activity both for membrane/apoplast and intracellular preparations (Bouzayen et al., 1990). All these studies used immunolocalization or activity assays in combination with (sub)cellular fractionations, and perhaps these techniques did not provide sufficient resolution to elucidate the exact ACO localization. A recent study tagged a safflower (*Carthamus tinctorius*) ACO with a GFP (green fluorescent protein) and performed an ectopic localization in onion epidermis cells. Their results showed that *Ct*ACO1 localizes in the cytosol (potentially linked with membranes) and in the nucleus, but their images lacked markers for these organelles (Tu et al., 2019). All studies combined are not conclusive about the exact ACO localization, and thus the actual subcellular site of ethylene production.

## ACO Phylogeny and Residue Analysis

The plant 2OGD superfamily can be categorized into three subclasses (DOXA, DOXB, and DOXC) based on amino acid sequence similarity (Kawai et al., 2014). ACO is a part of the DOXC subclass, the largest and most diverse group, containing over 400 different 2OGDs, mainly linked to the specialized metabolism (Kawai et al., 2014). Kawai et al. (2014) further subcategorized the DOXC subclass and classified ACO as part of the DOXC53 subclade. This subclade has 2OGD members, which are typically retrieved in all angiosperms. ACO is a unique member of the plant 2OGD superfamily, because it uses ascorbate as a catalyst instead of 2-oxoglutarate (Kawai et al., 2014).

A small phylogenetic study using a limited amount of ACO sequences from tomato (*Solanum lycopersicum*), potato (*Solanum tuberosum*), bonnet pepper (*Capsicum chinense*), petunia (*Petunia hybrida*), and tobacco (*Nicotiana tabacum*) classified the ACO protein family in three distinct phylogenetic groups (Jafari et al., 2013). A more detailed phylogenetic analysis of putative ACOs from mosses, lycophytes, gymnosperms, monocots, and dicots showed that ACO got more diversified after the monocot-dicot split (Clouse and Carraro, 2014). They also observed that there are three main clusters of ACOs and that monocot and dicot ACOs diverged together from a common pre-gymnosperm ancestor (Clouse and Carraro, 2014).

**FIGURE 2:**
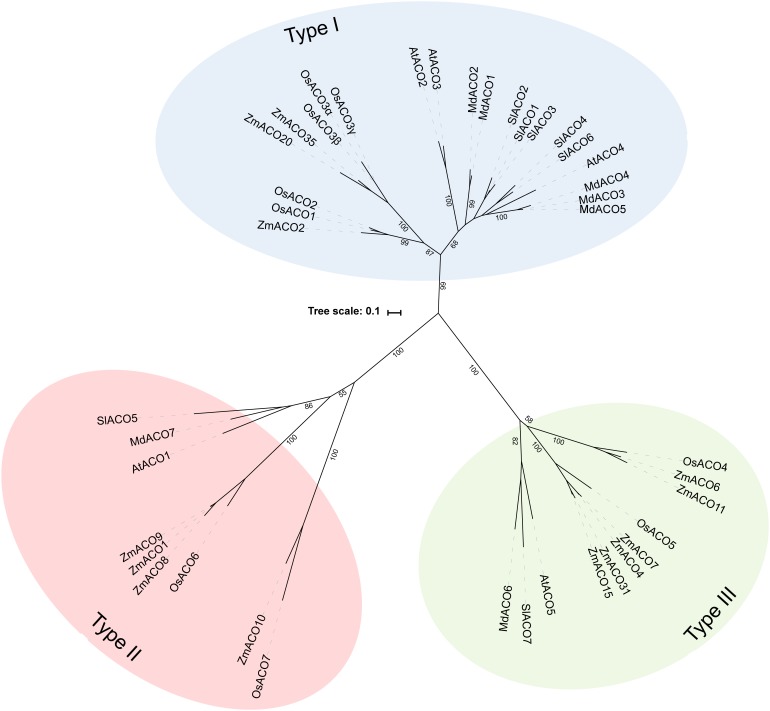
Maximal likelihood phylogenetic tree for ACO protein sequences of *Arabidopsis thaliana* (AT), Tomato (*Solanum lycopersicum*; Solyc), Apple (*Malus domestica*; MDP), Rice (*Oryza sativa*; Os), and Maize (*Zea mays*; Zm) retrieved from Phytozome (v12.1). Protein sequences were aligned in Geneious (v10.2.2) using the MUSCLE alignment plugin. The phylogenetic tree was build using RAxML (v8.2.11) for best-scoring maximum likelihood tree with rapid bootstrapping (1000 bootstrap replicates). Bootstrap values for the main branches are depicted on the tree. Type I ACO is shown in blue, Type II ACO is shown in red, and Type III ACO is shown in green.

Because not many ACOs have been shown to be functional ACO enzymes that can convert ACC into ethylene, it remains questionable if putative ACOs used in phylogenetic analyses are in fact functional ACOs. Trivial protein sequence similarity searches may lead to false or incorrect ACO annotations in genome and protein databases. In fact, there are only a few studies that have purified recombinant ACOs for functional characterization. This was done for tomato (*Sl*ACO1-3; Solyc07g049530, Solyc12g005940, Solyc07g049550; Bidonde et al., 1998), petunia (PhACO1; Zhang et al., 2004), apple (*Md*ACO1; MDP0000195885; Dilley et al., 2013), and Arabidopsis (*At*ACO2; AT1G62380; Sun et al., 2017). The study of Clouse and Carraro (2014) used annotated, but functionally unverified, ACO protein sequences as queries to identify novel ACO sequences in other species, without performing reciprocal BLAST searches. This approach resulted in the identification of false ACOs, leading to an overestimation of the size of the ACO protein family in certain species (e.g., 13 ACO members for *Arabidopsis thaliana* instead of 5). Therefore, we have performed a novel sequence similarity search using only the tomato ACO1 (Solyc07g049530) as search query, because this protein has been shown to be a true ACO with a confirmed activity (Bidonde et al., 1998). BLASTp jobs were done for 21 species using the Phytozome (v12.1.) database and Gymno plaza 1.0 (Proost et al., 2014), and top hits were only retained after a positive reciprocal BLAST search. Table 1 lists all the putative ACOs for some agriculturally important crops and Arabidopsis, while Supplementary Table 1 lists all the ACOs for the other plant species used in our phylogenetic analysis. We were able to identify 5 ACO members for Arabidopsis, 7 for tomato, 7 for apple, 9 for rice, and 13 for maize (Table 1). All putative ACO sequences were used to build a phylogenetic tree (see Supplementary Figure 1), which clearly shows a cluster of “ancient” ACOs within the clade of non-seed land plants and algae. This ancient clade most likely originated from an evolutionary distant algal 2OGD that gradually diverged into a functioning ACO during seed plant evolution. A more detailed phylogenetic tree of a selected amount of agriculturally important angiosperms shows 3 clusters of ACOs (Figure 2). Therefore, we suggest dividing the ACO family in three types: Type I, Type II, and Type III ACO. These three clusters are also observed in the larger phylogenetic tree of Supplementary Figure 1. Our analysis also shows that the gymnosperm ACOs group within the Type III ACO cluster of angiosperms, and that monocot and dicot ACOs diverged separately for each individual type. Our phylogenetic analysis indicates that the 3 types of ACOs diverged in parallel from a shared non-seed plant ancestral ACO or 2ODG.

**Table 1:** List of ACO sequences used for construction of the maximal likelihood phylogenetic tree of *Arabidopsis thaliana, Solanum lycopersicum, Malus domestica, Oryza sativa*, and *Zea mays*.

Species	Gene	GeneID	Type	Protein (aa)	Source
*Arabidopsis thaliana*	*AtACO1*	AT2G19590.1	2	311	Vandenbussche et al., 2003
	*AtACO2*	AT1G62380.1	1	321	Raz and Ecker, 1999
	*AtACO3*	AT1G12010.1	1	321	Vandenbussche et al., 2003
	*AtACO4*	AT1G05010.1	1	324	Gómez-Lim et al., 1993
	*AtACO5*	AT1G77330.1	3	308	Vandenbussche et al., 2003
Apple	*MdACO1*	MDP0000195885	1	314	Dong et al., 1992
(*Malus domestica*)	*MdACO2*	MDP0000200737	1	330	Binnie and McManus, 2009
	*MdACO3*	MDP0000725984	1	323	Binnie and McManus, 2009
	*MdACO4*	MDP0000251295	1	322	
	*MdACO5*	MDP0000453114	1	323	
	*MdACO6*	MDP0000025650	3	298	
	*MdACO7*	MDP0000200896	2	348	
Rice	*OsACO1*	LOC_Os09g27820.1	1	323	Chae et al., 2000
(*Oryza sativa*)	*OsACO2*	LOC_Os09g27750.1	1	323	Chae et al., 2000
	*OsACO3α*	LOC_Os02g53180.1	1	345	Chae et al., 2000
	*OsACO3β*	LOC_Os02g53180.2	1	322	Chae et al., 2000
	*OsACO3γ*	LOC_Os02g53180.3	1	284	Chae et al., 2000
	*OsACO6*	LOC_Os06g37590.1	2	294	
	*OsACO7*	LOC_Os01g39860.1	2	313	Iwai et al., 2006
	*OsACO4*	LOC_Os11g08380.1	3	310	Iwai et al., 2006
	*OsACO5*	LOC_Os05g05680.1	3	309	Iwai et al., 2006
Tomato	*SlACO1*	Solyc07g049530.2.1	1	316	Hamilton et al., 1991
(*Solanum lycopersicum*)	*SlACO2*	Solyc12g005940.1.1	1	317	Holdsworth et al., 1987
	*SlACO3*	Solyc07g049550.2.1	1	317	(Bidonde et al., 1998)
	*SlACO4*	Solyc02g081190.2.1	1	321	Nakatsuka et al., 1998
	*SlACO5*	Solyc07g026650.2.1	2	302	Sell and Hehl, 2005
	*SlACO6*	Solyc02g036350.2.1	1	320	
	*SlACO7*	Solyc06g060070.2.1	3	315	
Maize	*ZmACO20*	Zm00008a017510_T01	1	453	Gallie and Young, 2004
(*Zea mays*)	*ZmACO35*	Zm00008a023130_T01	1	304	Gallie and Young, 2004
	*ZmACO2*	Zm00008a028217_T01	1	327	
	*ZmACO8*	Zm00008a009058_T01	2	239	
	*ZmACO9*	Zm00008a021339_T01	2	235	
	*ZmACO1*	Zm00008a024831_T01	2	283	
	*ZmACO10*	Zm00008a031986_T01	2	319	
	*ZmACO11*	Zm00008a008130_T01	3	326	
	*ZmACO6*	Zm00008a018191_T01	3	314	
	*ZmACO31*	Zm00008a037498_T01	3	315	Gallie and Young, 2004
	*ZmACO4*	Zm00008a037500_T01	3	316	
	*ZmACO7*	Zm00008a037501_T01	3	296	
	*ZmACO15*	Zm00008a037502_T01	3	315	Gallie and Young, 2004


*Sequences were retrieved from Phytozome (v12.1) and top hits were only retained after a positive reciprocal BLAST search. Numbering of the different ACOs was done according to previously published work (if available), otherwise a new numbering is proposed. Putative splice variants of the same gene are represented by α, β, and γ.*

**FIGURE 3:**
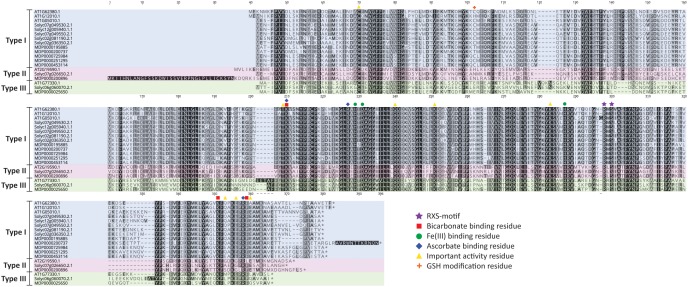
ACO protein sequence alignment of *Arabidopsis thaliana, Solanum lycopersicum*, and *Malus domestica*. Protein sequences were aligned in Geneious (v10.2.2) using the MUSCLE alignment plugin. Important residues are marked according to the legend shown. Type I ACO is shown in blue, Type II ACO is shown in red, and Type III ACO is shown in green.

A detailed residue analysis of the ACO alignment of Arabidopsis, tomato and apple presented in Figure 3 further confirms the existence of 3 types of ACO. The important 2-His-1-carboxylate Fe(II) binding motif is conserved in all ACOs. Shaw et al. (1996) provided some first experimental insight that this motif is composed of the H177-D179-H234 triad in *Md*ACO1 and that it is essential for ACO activity. This was confirmed in other studies in apple (Kadyrzhanova et al., 1999; Yoo et al., 2006) and for a petunia (Zhang et al., 2004) and tomato ACO (Brisson et al., 2012) (see also Supplementary Table 2).

Furthermore, a thorough mutagenesis study of *Md*ACO1, identified other important residues essential for ACO activity: C28, T157, K158, R175, Q188, K199, K230, R244, S246, K292, E294, E297, R299, F300, and E301 (Dilley et al., 2013). Some residues (R175, R299, and K158) have been proposed to coordinate bicarbonate binding (Zhang et al., 2004; Brisson et al., 2012; Dilley et al., 2013), while other residues (K292, K158, and F300) are proposed binding sites for ascorbate (Dilley et al., 2013). Besides these six amino acids, two additional residues (R244 and S246; highly conserved and part of the so-called RXS motif) complete the ACC/bicarbonate/ascorbate binding site of ACO (Kadyrzhanova et al., 1999; Seo et al., 2004; Zhang et al., 2004; Brisson et al., 2012; Dilley et al., 2013). Interestingly, the three ACO types can be classified based on the intermediate residue present in the conserved RXS-motif. This motif consists of R-M-S for type I ACOs, R-L/I-S for type II ACOs, and R-R-S for type III ACOs. All the residues considered important for ACO activity according to Dilley et al. (2013), are conserved in the three types of ACO, except for E294, E297, and E301. E294 is not well conserved in the three ACO types, while E297 is replaced by glycine only in the type II ACOs and E301 is not conserved in type III ACOs. It remains to be investigated whether or not the 3 types of ACOs actually have differences in functionality related to for example enzyme activity and/or protein stability.

## The Regulation of ACO

ACC-oxidase is expressed to a variable degree in all vegetative and reproductive tissues, which led to the belief that ACO proteins are always present and ready to produce ethylene. Furthermore, treating plant tissue with ACC typically results in a rapid production of ethylene. Therefore, it has been proposed that not ACO, but ACS is the rate-limiting enzyme in ethylene biosynthesis (Adams and Yang, 1979). This hypothesis has been readily absorbed by the community, leading to an abundance of studies focusing on unraveling the regulation and function of ACS in relation to its prime role in ethylene production (Argueso et al., 2007; Booker and DeLong, 2015; Yoon, 2015). However, there is an increasing amount of evidence demonstrating the importance of ACO, and not ACS, in controlling ethylene production in plants. For example ACO is the rate limiting step during flooding of tomato (English et al., 1995) and *Rumex palustris* (Vriezen et al., 1999). ACO activity, and not the availability of ACC, has been shown to be crucial during the formation of tension wood in poplar trees (Andersson-Gunneras et al., 2003; Love et al., 2009). Ethylene induced cotton fiber cell elongation has also been linked to a strong upregulation of its respective *ACO* genes (Shi et al., 2006). ACO has also been shown to be rate limiting during the post-climacteric ripening of tomato fruit (Van de Poel et al., 2012; Grierson, 2014; Van de Poel et al., 2014a,b). Even more recently, a key role for ACO during the sex determination of cucumber flowers was discovered (Chen et al., 2016). These studies indicate that ACO can sometimes be rate limiting and thus controls ethylene production, indicative of a stringent regulatory mechanism that controls ACO expression, stability and/or activity.

### Transcriptional Regulation of ACO

Despite the fact that *ACO* expression has been observed to be temporally and spatially regulated (e.g., during tomato flower and fruit development; Barry et al., 1996; Blume and Grierson, 1997; Nakatsuka et al., 1998; Van de Poel et al., 2012), only a few transcription factors have been identified that are known to control *ACO* expression (see Table 2). In tomato, *Sl*HB-1, a homeodomain-leucine zipper (HD-Zip) class-I transcription factor was shown to interact with the tomato *ACO1* (Solyc07g049530) promoter using gel retardation assays (Lin et al., 2008). Furthermore, experiments using virus-induced gene silencing showed that a repression of *HB-1* expression resulted in a decrease in *ACO1* transcript levels (Lin et al., 2008). Additionally, Lin et al. (2008) predicted that HB-1 could also target other ripening-related genes such as *ACO2* (Solyc12g005940), *PG1, RIN*, and *NOR* (Lin et al., 2008). Martel et al. (2011) reported that the master ripening regulator RIN could interact with the promoter of *HB-1*, placing HB-1 downstream of RIN during tomato fruit ripening. Later it was shown that RIN itself can interact directly with the *CArG* box in the promoter region of *ACO4* (Solyc02g081190) (Li et al., 2017). Besides HB-1 and RIN, different NAC transcription factors have also been observed to play an important role in the control of ethylene biosynthesis in tomato. Specifically, SNAC4 and SNAC9 have been shown to influence tomato fruit ripening by interacting with the promoters of *ACS2, ACS4*, and *ACO1* (Kou et al., 2016). Silencing *SNAC4* and *SNAC9* dramatically reduces the expression of these genes, inhibiting fruit ripening. Furthermore, silencing of *ERF2, ACS4*, and *ACO1* also reduces the expression of both *SNAC4* and *SNAC9*, which suggests the existence of a tightly controlled feedback mechanism (Kou et al., 2016).

**Table 2:** Functionally confirmed transcription factors that control *ACO* expression in *Solanum lycopersicum, Musa acuminata, Arabidopsis thaliana, Cucumis melo*, and *Cucumis sativus*.

Species	ACO target	Transcription factor	Source
Tomato (*Solanum lycopersicum*)	*ACO1*	HB-1	Lin et al., 2008
	*ACO4*	RIN	Li et al., 2017
	*ACO1*	NAC (SNAC9)	Kou et al., 2016
	*ACO3*	ERF2 and TERF2	Zhang et al., 2009
Banana (*Musa acuminata*)	*ACO1*	ERF11	Han et al., 2016
	*ACO1*	MADS7	Liu et al., 2015
*Arabidopsis thaliana*	*ACO5*	SHYG	Rauf et al., 2013
Melon (*Cucumis melo*)	*ACO1*	EIL1 and EIL2	Huang et al., 2010
	*ACO3*	WIP1	Chen et al., 2016
Cucumber (*Cucumis sativus*)	*ACO2*	WIP1	Chen et al., 2016


Ethylene response factors (ERFs) have been shown to be an integral part of the ethylene signaling and response pathway. ERFs are transcription factors that can bind with *cis*-acting elements such as GCC-box motifs and dehydration-responsive elements (DREs) (Ohme-Takagi and Hideaki, 1995; Müller and Munné-Bosch, 2015). Zhang et al. (2009) showed that the tomato *ERF2* (and a homolog allele *TERF2*) was able to interact with the DRE in the promoter of *SlACO3* to activate transcription. They observed a significant increase in ethylene production of the *ERF2/TERF2* overexpression lines and a decrease in the *ERF2/TERF2* antisense-lines compared to the wild type, suggesting that these ERFs are positive regulators of *ACO3* expression in tomato (Zhang et al., 2009).

In banana (*Musa acuminata*), the transcription factor ERF11 was shown to interact directly with the GCC-box motif in the promoter region of *ACO1* and repress *ACO1* expression (Han et al., 2016). Han et al. (2016) also demonstrated that ERF11 can physically interact with the histone deacetylase HDA1, which in turn reinforces the ERF11-induced repression of *ACO1.* Furthermore, the MADS-box transcription factor MADS7 was also shown to interact directly with the promoter of *ACO1* in banana using a yeast one-hybrid (Y1H) system and a transient GUS-reporter activation assay in tobacco (Liu et al., 2015). *MADS7* is only expressed in banana fruit and its expression is stimulated by ethylene and inhibited by 1-MCP. Ectopic overexpression of *MaMADS7* in tomato fruit resulted in a 10-fold increase of *SlACO1* expression compared to wild-type fruit, and resulted in an enhanced ethylene production level (Liu et al., 2015).

Another transcription factor that controls *ACO* expression was also identified in melon fruit (*Cucumis melo*). Huang et al. (2010) reported that EIN3-like proteins EIL1 and EIL2 induce the expression of *ACO1* by interacting with different *cis*-acting elements of the *ACO1* promoter. It was hypothesized that both EIL proteins are targeted for proteolysis by EBF1/EBF2 (similar as in Arabidopsis) in the absence of ethylene, however, upon ethylene release they are stabilized and elevate the biosynthesis of ethylene by inducing the transcription of *ACO1* and thus promote ripening (Huang et al., 2010).

In cucumber (*Cucumis sativus*), the transcription factor WIP1 can regulate flower sex determination by directly binding the promoter of *ACO2* and inhibiting its expression (Chen et al., 2016). Evidence was provided using a dual luciferase activation assay in tobacco, Y1H, ChIP-qPCR, and EMSA to validate the interaction between WIP1 and the *ACO2* promoter (Chen et al., 2016). Chen et al. (2016) also demonstrated that the melon (*Cucumis melo*) homolog of WIP1 can interact with the promoter of *CmACO3*, and similarly as in cucumber, negatively influence *CmACO3* expression.

In *Arabidopsis thaliana*, the NAC transcription factor Speedy Hyponastic Growth (SHYG) was shown to interact with the promoter region of *ACO5* (AT77330; Rauf et al., 2013). When *SHYG* was overexpressed using an inducible promoter, the expression of *ACO5* was shown to be strongly induced (Rauf et al., 2013).

#### Differential *ACO* Expression Profiles

In order to get a better insight in the differential expression of the *ACO* gene family, we have summarized the tissue-specific and developmental expression profiles for Arabidopsis and tomato using the eFP browser (http://bar.utoronto.ca/efp/cgi-bin/efpWeb.cgi; Waese et al., 2017) and the Tomato Expression Atlas (http://tea.solgenomics.net/), respectively.

##### Differential ACO expression in Arabidopsis thaliana

**FIGURE 4:**
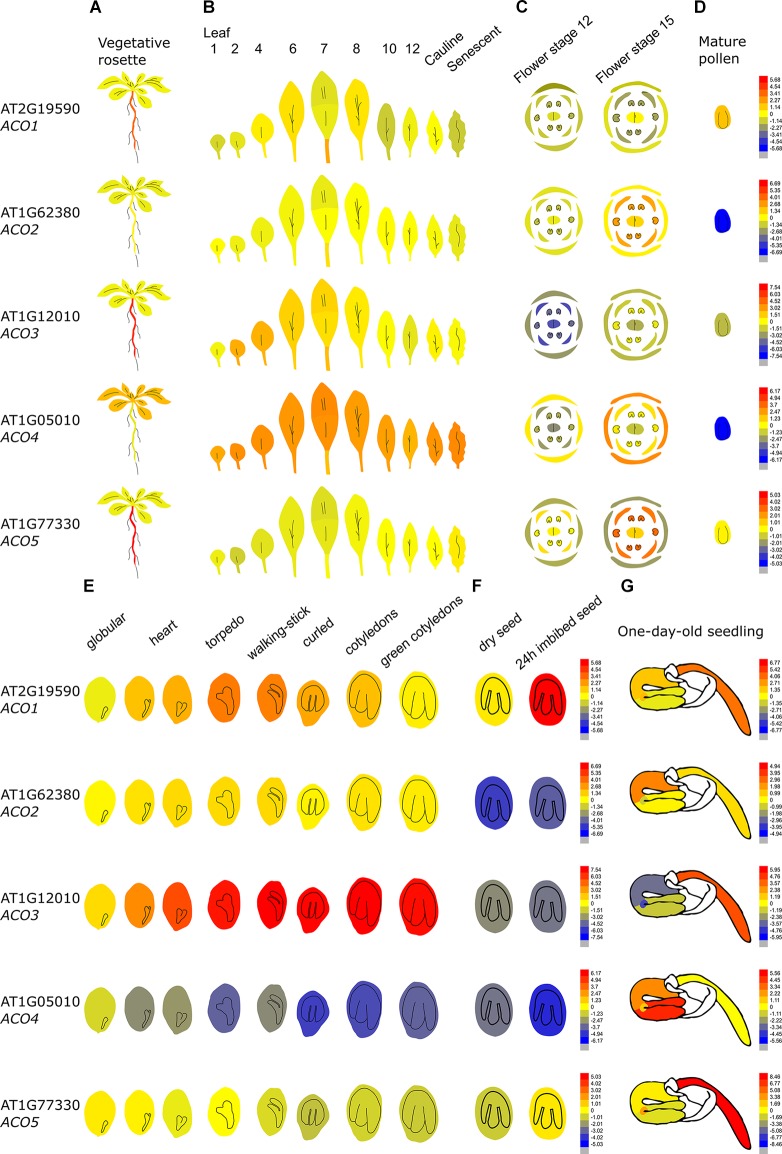
Tissue-specific and developmental expression profiles of the five Arabidopsis ACOs. Data is adapted from the eFP browser. The color scale depicts a Log2 fold expression range (Waese et al., 2017). **(A)** Expression in the vegetative rosette and the root, **(B)** the different leaves, **(C)** the different flowering organs, **(D)** mature pollen, **(E)** the different stages of embryogenesis, **(F)** the dry and imbibed seed, and **(G)** a 1-day-old seedling (Nakabayashi et al., 2005; Schmid et al., 2005; Klepikova et al., 2016).

Figure 4 demonstrates that the *Arabidopsis thaliana ACO* genes show a distinct tissue-specific expression pattern. *ACO1* (AT2G19590; Type II) is upregulated during the torpedo and walking-stick stage of embryogenesis, is highly expressed in imbibed seeds and upregulated during the first stages of germination, mainly in the radicle. Furthermore, *ACO1* is also strongly expressed in the roots, where it might be involved in lateral root formation (Park et al., 2018). *ACO2* (AT1G62380; Type I) was shown to be involved in germination, where it participates in ethylene production to control endosperm cap weakening and endosperm rupture (Linkies et al., 2009; Linkies and Leubner-Metzger, 2012). *ACO2* is mostly expressed in the emerging seedling hypocotyl, where it is involved in the formation of the apical hook (Raz and Ecker, 1999). *ACO2* is also highly expressed in the phloem and companion cells of the roots (not shown in Figure 4; Brady et al., 2007). *ACO2* is also upregulated during flower opening and specifically during anther, stamen and petal development (van Es et al., 2018). *ACO3* (AT1G12010; Type I) is highly expressed during embryogenesis and during further seed maturation. Furthermore, *ACO3* is expressed in the root, more precisely in the phloem and companion cells (Brady et al., 2007). *ACO4* (AT1G05010; Type I) is mostly expressed in vegetative tissue such as the cotyledons, the rosette, cauline leaves, sepals, and the petiole of senescing leaves. *ACO5* (AT177330; Type III), is mainly expressed in the root (of both seedlings and adult plants), especially in the root apex and the root cap (Brady et al., 2007).

##### Differential ACO expression during tomato fruit development and climacteric ripening

**FIGURE 5:**
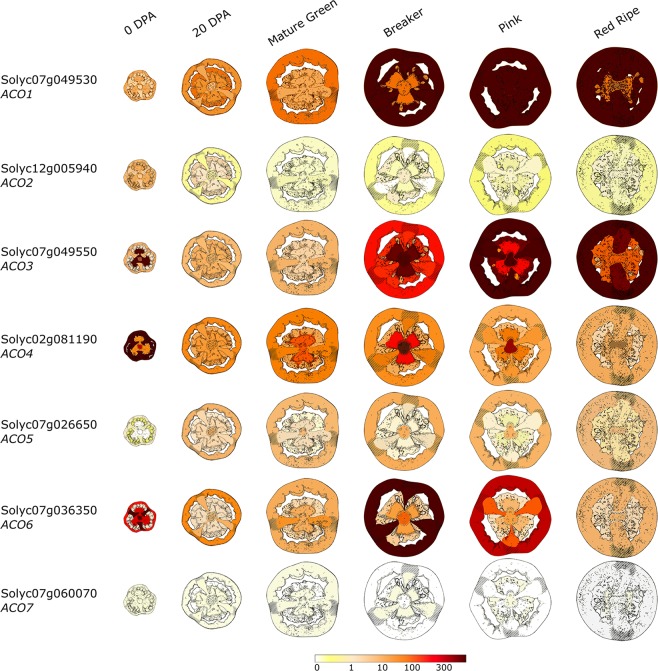
Tissue-specific and developmental expression profiles of the seven tomato ACOs during fruit development and ripening. Data is adapted from the Tomato Expression Atlas (TEA). The color scale depicts the reads per million mapped reads (RPM). Days post anthesis (DPA).

Figure 5 summarizes the differential and tissue-specific expression of the seven *ACOs* during tomato fruit development and climacteric ripening, based on the Tomato Expression Atlas (TEA). *ACO1* (Solyc07g049530; Type I) is already expressed shortly after anthesis, and expression levels increase moderately throughout fruit development (system I). At the onset of ripening (system II), *ACO1* expression increases strongly and correlates well with the autocatalytic rise in ethylene production (Blume and Grierson, 1997; Nakatsuka et al., 1998; Alexander and Grierson, 2002; Van de Poel et al., 2012). *ACO1* expression appears to be strongest in the pericarp, septa and columella of orange to red fruit, matching the tissue-specific ACO *in vitro* activity reported by Van de Poel et al. (2014b). *ACO2* (Solyc12g005940; Type I) is expressed at anthesis, however, expression drops to a basal level during further fruit development and ripening (Van de Poel et al., 2012). Expression of *ACO3* (Solyc07g049550; Type I) is high during anthesis, but readily drops during initial fruit development. At the onset of ripening, *ACO3* expression strongly increases again. These observations are contradictory to the qPCR data observed by Van de Poel et al. (2012), who showed that *ACO3* expression declines after the breaker stage. *ACO4* (Solyc02g081190; Type I) expression is high during initial fruit development (mainly pericarp tissue) and declines thereafter to a basal expression level. However, there is a temporal increase in *ACO4* expression during the breaker stage, mainly in the columella and placenta tissue. Nakatsuka et al. (1998) showed that *ACO4* expression increases during fruit ripening, but perhaps the use of degenerate primers in the Nakatsuka study could not discriminate between *ACO4* and another *ACO* homolog. *ACO5* (Solyc07g026650; Type II) expression increases slightly after anthesis and remains at a similar level during further fruit development and ripening. However, qPCR analysis by Van de Poel et al. (2012) indicated that *ACO5* follows an expression pattern similar to that of *ACO3*. *ACO6* (Solyc02g036350; Type I) is strongly expressed at anthesis, followed by a low expression during fruit development and a temporal high expression during the breaker stage, followed by a gradual decline during further ripening. *ACO6* expression is strongest in the pericarp tissue, which is also the tissue that showed the highest *in vivo* ethylene production (Van de Poel et al., 2014b). *ACO7* (Solyc06g060070; Type III) is only basally expressed during fruit development and ripening. The *ACO6* and *ACO7* genes have not yet been characterized during tomato fruit development and ripening.

### Post-transcriptional Regulation of ACO

MicroRNAs (miRNAs) are often involved in the post-transcriptional regulation of diverse processes in plant growth and development. MiRNAs constitute a class of regulatory, small, non-coding RNA molecules of 20 to 24 nucleotides long, which can intervene in gene expression by cleaving mRNA transcripts in a sequence specific way (Liu et al., 2017, 2018; Yu et al., 2017). MiR396b was identified as a cold-responsive miRNA in the cold hardy citrus variety *Ponciferus trifoliata* (Zhang et al., 2014). When the precursor of this miRNA (MIR396b) was overexpressed in lemon (*Citrus lemon*), it led to an increase in cold tolerance. Interestingly, overexpression of this miRNA reduced the expression of *ACO* compared to the wild type lemon. Zhang et al. (2016) showed that miR396b directs the cleavage of *ACO* transcripts, consequently inhibiting ethylene biosynthesis.

Recently, a second miRNA was identified, which affects the expression of *ACO* (Wang et al., 2018). In tomato, *miR1917* directs the cleavage of a specific *CTR4* splice variant in tomato leading to an altered ethylene response. Overexpression of *miR1917* significantly enhanced the expression of *ACS2, ACS4, ACO1* (Solyc07g049530) and *ACO3* (Solyc07g049550), leading to specific ethylene response phenotypes such as the triple response in etiolated seedlings, an increase in epinastic curvature of leaf petioles, an increased pedicel abscission rate and an accelerated fruit ripening (Wang et al., 2018).

### Post-translational Regulation of ACO

An *in vitro* phosphorylation assay using protein extracts of pre- and post-climacteric apple fruit and an ectopically expressed His-tagged *Md*ACO1 (MDP0000195885) hinted for the first time that ACO protein-protein interactions could exist (Dilley et al., 1995). It was observed that apple ACO1 could interact with unidentified proteins and resulted in the phosphorylation of these associated proteins (and possibly also ACO1 itself) in both pre-and post-climacteric samples (Dilley et al., 1995). Later Dilley et al. also speculated about a possible cysteine protease activity of ACO, unrelated to its role in catalyzing the conversion of ACC to ethylene. This hypothesis was based on sequence similarity between ACO and a tomato cysteine protease (Matarasso et al., 2005), and the strongly conserved C28 as a key residue in the active site of this protease (Dilley et al., 2013). Using *in silico* predictions based on sequence similarity, other motifs for ACO protein-protein interactions, serine/threonine kinases, tyrosine kinases, and glycosylation were identified (Aitken, 1999; Dilley et al., 2013). Despite these predictions, only a few post-translational modifications of ACO have currently been experimentally observed.

Arabidopsis ACO2 (AT1g62380) was identified as a target in a broad proteomics screen that characterized stress-induced protein *S*-glutathionylation, suggesting that ACO2 is post-translationally modified by glutathionylation (Dixon et al., 2005). This was later confirmed in a dedicated S-glutathione pull-down assay with ACO2, where C63 was identified as the target residue for glutathionylation (Datta et al., 2015).The relevance of this post-translational glutathionylation and its involvement in ethylene production remains unknown.

Another thiol-residue modification of ACO was discovered in a large cysteine S-sulfhydration screen in Arabidopsis, which showed that ACO4 can get sulfhydrated (Aroca et al., 2015). This S-modification of ACO was recently confirmed in tomato by Jia et al. (2018), who demonstrated that H_2_S production increases in stomata upon a prolonged ethylene treatment, and that this leads to the S-sulfhydration of C60 (equivalent to C63 of *At*ACO2) of *Sl*ACO1 (Solyc07g049530) and *Sl*ACO2 (Solyc12g005940). This ACO sulfhydration resulted in a significant drop in ACO activity and consequently ethylene production, unmasking a direct crosstalk between ethylene and H_2_S production. It was argued that this sulfhydration was necessary to protect the plant from the detrimental effects of prolonged ethylene production and exposure (e.g., during senescence and programmed cell death) (Jia et al., 2018). At the moment, it remains unclear if this cysteine glutathionylation and/or sulfhydration leads to a different ACO activity or whether it is involved in protein interaction and/or protein stability. In general, post-translational modifications of the thiol-groups of cysteine residues by S-glutathionylation or S-sulfhydration are involved in the protection of proteins from irreversible oxidation or redox changes (Gao et al., 2009; Datta et al., 2015) or modulate protein-protein interactions (Aroca et al., 2015). The exact function of ACO S-glutathionylation and S-sulfhydration remains to be further investigated.

In petunia, Tan et al. (2014) identified GRL2 (Green-like 2) as a novel interacting partner of ACO1 in a yeast two-hybrid screen using GRL2 as bait and a cDNA library of petals and leaves as prey. They also observed that the suppression of *GRL2* expression resulted in an increased ethylene production of flowers, leading to an accelerated flower senescence. Therefore, GRL2 is proposed to serve as a negative regulator of ethylene production that can directly influence the activity of ACO1 (Tan et al., 2014).

## ACO Biotechnology and Applications

Because ethylene plays a crucial role in many plant processes, including climacteric fruit ripening and senescence, excessive ethylene can lead to unwanted decay of plant-based food. Therefore, ethylene biosynthesis or signaling genes have been frequently targeted in biotechnological and transgenic approaches in order to increase the shelf life of plant-based food. Because ACO catalyzes the final step in the ethylene biosynthesis pathway, it is therefore an ideal candidate to target (instead of for example ACS or ethylene signaling components), because there are fewer risk of intervening in other pathways (e.g., ACC metabolism). Table 3 presents an exhaustive list, although probably not exclusive, of several transgenic applications controlling ethylene production at the level of ACO for important agricultural crops.

**Table 3:** Overview of ACO-directed transgenic applications in different agricultural crops.

Crop	Target gene	Approach	References
Apple (*Malus domestica*)	*ACO1 + ACS 8-like*	Antisense RNA	Dandekar et al., 2004
Broccoli (*Brassica oleracea*)	*ACO*	Antisense RNA	Henzi et al., 1999
	*ACO2*	Antisense RNA	Gapper et al., 2005
Carnation (*Dianthus caryophyllus*)	*ACO*	Antisense RNA	Savin et al., 1995
Lemon (*Citrus lemon*)	MIR396b	Overexpression	Zhang et al., 2016
Kiwi (*Actinidia chinensis*)	*ACO1-4 + ACO6*	RNAi	Atkinson et al., 2011
Melon (*Cucumis melo*)	*ACO1*	Antisense RNA	Ayub et al., 1996; Bauchot et al., 1998; Ben-Amor et al., 1999
	*ACO*	Antisense RNA	Guis et al., 1997
	*ACO*	Antisense RNA	Silva et al., 2004
	*ACO1*	Antisense RNA	Nuñez-Palenius et al., 2006
Papaya (*Carica papaya*)	*ACO1*	Cosuppression	López-Gómez et al., 2009
	*ACO1 + ACO2*	RNAi	Sekeli et al., 2014
Poplar	*ACO1*	Overexpression	Love et al., 2009
Pear (*Pyrus communis*)	*ACO1*	Sense/antisense RNA	Gao et al., 2007
Petunia	*ACO + ACS*	Antisense RNA	Huang et al., 2007
	*GRL2*	VIGS	Tan et al., 2014
Safflower (*Carthamus tinctorius*)	*ACO1*	Overexpression	Tu et al., 2019
Tobacco (*Nicotiana tabacum*)	*ACO*	Sense/antisense RNA	Knoester et al., 1997
Tomato (*Solanum lycopersicum*)	*ACO1*	Antisense RNA	Hamilton et al., 1990; Picton et al., 1993
	*ACO1*	Antisense RNA	English et al., 1995
	*ACO*	Antisense RNA	Batra et al., 2010
	*ACO*	RNAi	Xiong et al., 2005
	*HD1*	VIGS	Lin et al., 2008
Torenia (*Torenia fournieri*)	*ACO*	Sense/antisense RNA	Aida et al., 1998


One of the most rewarding applications of ACO-directed biotechnology in plants is the reduction of ethylene production during ripening and postharvest storage of climacteric fruit. *ACO* has been targeted using various antisense RNAi techniques to downregulate its expression and consequently decrease ethylene production, resulting in control over fruit ripening and postharvest storage. Table 3 shows that this approach was successfully implemented for a wide variety of fruits: apple, lemon, kiwi, melon, papaya, pear, and tomato (Hamilton et al., 1990; Picton et al., 1993; English et al., 1995; Ayub et al., 1996; Guis et al., 1997; Bauchot et al., 1998; Ben-Amor et al., 1999; Dandekar et al., 2004; Silva et al., 2004; Xiong et al., 2005; Nuñez-Palenius et al., 2006; Gao et al., 2007; Lin et al., 2008; López-Gómez et al., 2009; Batra et al., 2010; Atkinson et al., 2011; Sekeli et al., 2014; Zhang et al., 2016). In all these studies, fruit ripening was delayed and shelf-life was prolonged. A similar approach has also been used to prolong the shelf life of vegetative tissue of vegetables, such as for example broccoli (Henzi et al., 1999; Gapper et al., 2005).

Transgenic approaches that silence *ACO* expression are also beneficial in floriculture. It has been shown that a reduced *ACO* expression resulted in a delay in flower senescence and flower abscission in petunia, carnation, and torenia (Savin et al., 1995; Aida et al., 1998; Huang et al., 2007; Tan et al., 2014). Besides controlling fruit ripening or flower senescence, *ACO*s have also been targeted in other ethylene-related processes. For example, tomato plants transformed with an *ACO1* antisense construct showed delayed leaf senescence (John et al., 1995) and less epinasty during soil flooding (English et al., 1995). A mutation in cucumber *ACO2* resulted in a mutant that only bears male flowers, suggesting that *ACO2* plays an important role in sex determination in cucumber flowers (Chen et al., 2016).

Besides silencing *ACO* expression and reducing ethylene production, it can sometimes be desirable to boost ethylene production, and then an *ACO* overexpression construct is most suitable. In safflower, *ACO1* overexpression was shown to stimulate the flavonoid biosynthesis pathway, which could be interesting for oilseed production (Tu et al., 2019). Overexpression of *ACO1* in poplar (*Populus tremula × tremuloides*) caused a stimulation of cambial cell division, which in turn resulted in an increased xylem development and an inhibition of elongation growth, which are desirable traits for the wood industry (Love et al., 2009). Altogether, these transgenic examples show the potential of controlling ethylene production levels by targeting the *ACO* gene family. Perhaps new breeding technologies such as CRISPR/Cas9-mediated mutations in the *ACO* promotor or coding sequence could also lead to novel strategies to control ethylene production in plants.

## Data Availability

No datasets were generated or analyzed for this study.

## Author Contributions

Both authors have made a substantial, direct and intellectual contribution to the work, and approved it for publication.

## Conflict of Interest Statement

The authors declare that the research was conducted in the absence of any commercial or financial relationships that could be construed as a potential conflict of interest.

## References

[B1] Adams D. O. Yang S. F. (1977). Methionine metabolism in apple tissue: implication of s-adenosylmethionine as an intermediate in the conversion of methionine to ethylene. *Plant Physiol.* 60 892–896. 10.1104/pp.60.6.892 16660208 PMC542741

[B2] Adams D. O. Yang S. F. (1979). Ethylene biosynthesis: Identification of 1-aminocyclopropane-1-carboxylic acid as an intermediate in the conversion of methionine to ethylene. *Proc. Natl. Acad. Sci. U.S.A.* 76 170–174. 10.1073/pnas.76.1.170 16592605 PMC382898

[B3] Adams D. O. Yang S. F. (1981). Ethylene the gaseous plant hormone: mechanism and regulation of biosynthesis. *Trends Biochem. Sci.* 6 161–164. 10.1016/0968-0004(81)90059-90051

[B4] Aida R. Yoshida T. Ichimura K. Goto R. Shibata M. (1998). Extension of flower longevity in transgenic torenia plants incorporating ACC oxidase transgene. *Plant Sci.* 138 91–101. 10.1016/S0168-9452(98)00139-133

[B5] Aik W. S. Chowdhury R. Clifton I. J. Hopkinson R. J. Leissing T. McDonough M. A. (2015). Introduction to structural studies on 2-oxoglutarate-dependent oxygenases and related enzymes. *RSC Metallobiol.* 2015 59–94. 10.1039/9781782621959-00059

[B6] Aitken A. (1999). Protein consensus sequence motifs. *Mol. Biotechnol.* 12 241–254. 10.1385/mb:12:3:241 10631681

[B7] Alexander L. Grierson D. (2002). Ethylene biosynthesis and action in tomato: a model for climacteric fruit ripening. *J. Exp. Bot.* 53 2039–2055. 10.1093/jxb/erf072 12324528

[B8] Amrhein N. Schneebeck D. Skorupka H. Tophof S. Stöckigt J. (1981). Identification of a major metabolite of the ethylene precursor 1-aminocyclopropane-1-carboxylic acid in higher plants. *Naturwissenschaften* 68 619–620. 10.1007/BF00398617

[B9] Andersson-Gunneras S. Hellgren J. M. Bjorklund S. Regan S. Moritz T. Sundberg B. (2003). Asymmetric expression of a poplar ACC oxidase controls ethylene production during gravitational induction of tension wood. *Plant J.* 34 339–349. 10.1046/j.1365-313X.2003.01727.x 12713540

[B10] Argueso C. T. Hansen M. Kieber J. J. (2007). Regulation of ethylene biosynthesis. *J. Plant Growth Regul.* 26 92–105. 10.1007/s00344-007-0013-5

[B11] Aroca Á Serna A. Gotor C. Romero L. C. (2015). S -Sulfhydration: a cysteine posttranslational modification in plant systems. *Plant Physiol.* 168 334–342. 10.1104/pp.15.00009 25810097 PMC4424021

[B12] Atkinson R. G. Gunaseelan K. Wang M. Y. Luo L. Wang T. Norling C. L. (2011). Dissecting the role of climacteric ethylene in kiwifruit (Actinidia chinensis) ripening using a 1-aminocyclopropane-1-carboxylic acid oxidase knockdown line. *J. Exp. Bot.* 62 3821–3835. 10.1093/jxb/err063 21511911

[B13] Ayub R. Guis M. Amor M. Ben Gillot L. Roustan J.-P. Latché A. (1996). Expression of ACC oxidase antisense gene inhibits ripening of cantaloupe melon fruits. *Nat. Biotechnol.* 14 862–866. 10.1038/nbt0796-862 9631011

[B14] Barry C. S. Blume B. Bouzayen M. Cooper W. Hamilton A. J. Grierson D. (1996). Differential expression of the 1-aminocyclopropane-1-carboxylate oxidase gene family of tomato. *Plant J.* 9 525–535. 10.1046/j.1365-313X.1996.09040525.x 8624515

[B15] Batra A. Sane V. A. Trivedi P. K. Sane A. P. Nath P. (2010). Suppression of ACC oxidase expression in tomato using heterologous gene from banana prolongs shelf-life both on vine and post-harvest. *Curr. Sci.* 99 1243–1250.

[B16] Bauchot A. D. Mottram D. S. Dodson A. T. John P. (1998). Effect of aminocyclopropane-1-carboxylic acid oxidase antisense gene on the formation of volatile esters in cantaloupe charentais melon (*Cv. Védrandais*). *J. Agric. Food Chem.* 46 4787–4792. 10.1021/jf980692z

[B17] Ben-Amor M. Flores B. Latché A. Bouzayen M. Pech J. C. Romojaro F. (1999). Inhibition of ethylene biosynthesis by antisense ACC oxidase RNA prevents chilling injury in *Charentais cantaloupe* melons. *Plant. Cell Environ.* 22 1579–1586. 10.1046/j.1365-3040.1999.00509.x

[B18] Bidonde S. Ferrer M. A. Zegzouti H. Ramassamy S. Latche A. Pech J.-C. (1998). Expression and characterization of three tomato 1-aminocyclopropane-1-carboxylate oxidase cDNAs in yeast. *Eur. J. Biochem.* 253 20–26. 10.1046/j.1432-1327.1998.2530020.x 9578456

[B19] Binnie J. E. McManus M. T. (2009). Characterization of the 1-aminocyclopropane-1-carboxylic acid (ACC) oxidase multigene family of *Malus* domestica borkh. *Phytochemistry* 70 348–360. 10.1016/j.phytochem.2009.01.002 19223050

[B20] Blume B. Grierson D. (1997). Expression of ACC oxidase promoter-GUS fusions in tomato and Nicotiana plumbaginifolia regulated by developmental and environmental stimuli. *Plant J.* 12 731–746. 10.1046/j.1365-313X.1997.12040731.x 9375389

[B21] Boller T. Herner R. C. Kende H. (1979). Assay for and enzymatic formation of an ethylene precursor, 1-aminocyclopropane-1-carboxylic acid. *Planta* 145 293–303. 10.1007/BF00454455 24317737

[B22] Booker M. A. DeLong A. (2015). Producing the ethylene signal: regulation and diversification of ethylene biosynthetic enzymes. *Plant Physiol.* 169 42–50. 10.1104/pp.15.00672 26134162 PMC4577410

[B23] Bouzayen M. Felix G. Latché A. Pech J.-C. Boller T. (1991). Iron: an essential cofactor for the conversion of 1-aminocyclopropane-1-carboxylic acid to ethylene. *Planta* 184 244–247. 10.1007/BF00197953 24194076

[B24] Bouzayen M. Latché A. Pech J.-C. (1990). Subcellular localization of the sites of conversion of 1-aminocyclopropane-1-carboxylic acid into ethylene in plant cells. *Planta* 180 175–180. 10.1007/BF00193992 24201941

[B25] Brady S. M. Mace D. Ohler U. Benfey P. N. (2007). A high-resolution root spatiotemporal map reveals spatiotemporal map reveals dominant expression patterns dominant expression patterns. *Science* 318 801–806. 10.1126/science.1146265 17975066

[B26] Brisson L. El Bakkali-Taheri N. Giorgi M. Fadel A. Kaizer J. Réglier M. (2012). 1-Aminocyclopropane-1-carboxylic acid oxidase: insight into cofactor binding from experimental and theoretical studies. *J. Biol. Inorg. Chem.* 17 939–949. 10.1007/s00775-012-0910-3 22711330

[B27] Burg S. P. Burg E. A. (1965). Ethylene action and the ripening of fruits. *Science* 148 1190–1196. 10.1126/science.148.3674.1190 14280001

[B28] Bürstenbinder K. Waduwara I. Schoor S. Moffatt B. A. Wirtz M. Minocha S. C. (2010). Inhibition of 5′-methylthioadenosine metabolism in the Yang cycle alters polyamine levels, and impairs seedling growth and reproduction in Arabidopsis. *Plant J.* 62 977–988. 10.1111/j.1365-313X.2010.04211.x 20345605

[B29] Chae H. S. Cho Y. G. Park M. Y. Lee M. C. Eun M. Y. Kang B. G. (2000). Hormonal cross-talk between auxin and ethylene differentially regulates the expression of two members of the 1-aminocyclopropane-1-carboxylate oxidase gene family in rice (*Oryza sativa* L.). *Plant Cell Physiol.* 41 354–362. 10.1093/pcp/41.3.354 10805599

[B30] Chen H. Sun J. Li S. Cui Q. Zhang H. Xin F. (2016). An ACC oxidase gene essential for cucumber carpel development. *Mol. Plant* 9 1315–1327. 10.1016/j.molp.2016.06.018 27403533

[B31] Chung M.-C. Chou S.-J. Kuang L.-Y. Charng Y. Yang S. F. (2002). Subcellular localization of 1-aminocyclopropane-1-carboxylic acid oxidase in apple fruit. *Plant Cell Physiol.* 43 549–554. 10.1093/pcp/pcf067 12040102

[B32] Clouse R. M. Carraro N. (2014). A novel phylogeny and morphological reconstruction of the PIN genes and first phylogeny of the ACC-oxidases (ACOs). *Front. Plant Sci.* 5:296. 10.3389/fpls.2014.00296 25018760 PMC4071234

[B33] Dandekar A. M. Teo G. Defilippi B. G. Uratsu S. L. Passey A. J. Kader A. A. (2004). Effect of down-regulation of ethylene biosynthesis on fruit flavor complex in apple fruit. *Transgenic Res.* 13 373–384. 10.1023/B:TRAG.0000040037.90435.45 15517996

[B34] Datta R. Kumar D. Sultana A. Hazra S. Bhattacharyya D. Chattopadhyay S. (2015). Glutathione regulates ACC synthase transcription via WRKY33 and ACC oxidase by modulating mRNA stability to induce ethylene synthesis during stress. *Plant Physiol.* 169 2963–2981. 10.1104/pp.15.01543 26463088 PMC4677924

[B35] Dilley D. R. Kuai J. Wilson I. D. Pekker Y. Zhu Y. Burmeister D. M. (1995). Molecular biological investigations of ACC oxidase and its expression attending apple fruit ripening. *Acta Hortic* 379 25–40. 10.17660/ActaHortic.1995.379.1

[B36] Dilley D. R. Wang Z. Kadirjan-Kalbach D. K. Ververidis F. Beaudry R. Padmanabhan K. (2013). 1-Aminocyclopropane-1-carboxylic acid oxidase reaction mechanism and putative post-translational activities of the ACCO protein. *AoB Plants* 5 1–23. 10.1093/aobpla/plt031 24244837 PMC3828642

[B37] Dixon D. P. Skipsey M. Grundy N. M. Edwards R. (2005). Stress-induced protein S-glutathionylation in *Arabidopsis*. *Plant Physiol.* 138 2233–2244. 10.1104/pp.104.058917 16055689 PMC1183410

[B38] Dong J. G. Olson D. Silverstone A. Yang S. F. (1992). Sequence of a cDNA coding for a 1-aminocyclopropane-1-carboxylate oxidase homolog from apple fruit. *Plant Physiol.* 98 1530–1531. 10.1104/pp.98.4.1530 16668829 PMC1080386

[B39] English P. J. Lycett G. W. Roberts J. A. Jackson M. B. (1995). Increased 1-aminocyclopropane-1-carboxylic acid oxidase activity in shoots of flooded tomato plants raises ethylene production to physiologically active levels. *Plant Physiol.* 109 1435–1440. 10.1104/pp.109.4.1435 12228680 PMC157679

[B40] Farrow S. C. Facchini P. J. (2014). Functional diversity of 2-oxoglutarate/Fe(II)-dependent dioxygenases in plant metabolism. *Front. Plant Sci.* 5:524. 10.3389/fpls.2014.00524 25346740 PMC4191161

[B41] Gallie D. R. Young T. E. (2004). The ethylene biosynthetic and perception machinery is differentially expressed during endosperm and embryo development in maize. *Mol. Genet. Genomics* 271 267–281. 10.1007/s00438-004-0977-9 14760521

[B42] Gao M. Matsuta N. Murayama H. Toyomasu T. Mitsuhashi W. Dandekar A. M. (2007). Gene expression and ethylene production in transgenic pear (Pyrus communis cv. ‘La France’) with sense or antisense cDNA encoding ACC oxidase. *Plant Sci.* 173 32–42. 10.1016/j.plantsci.2007.03.014

[B43] Gao X.-H. Bedhomme M. Veyel D. Zaffagnini M. Lemaire S. D. (2009). Methods for analysis of protein glutathionylation and their application to photosynthetic organisms. *Mol. Plant* 2 218–235. 10.1093/mp/ssn072 19825609

[B44] Gapper N. E. Coupe S. A. McKenzie M. J. Scott R. W. Christey M. C. Lill R. E. (2005). Senescence-associated down-regulation of 1-aminocyclopropane-1-carboxylate (ACC) oxidase delays harvest-induced senescence in broccoli. *Funct. Plant Biol.* 32:891 10.1071/FP05076 32689185

[B45] Gómez-Lim M. A. Valdés-López V. Cruz-Hernandez A. Saucedo-Arias L. J. (1993). Isolation and characterization of a gene involved in ethylene biosynthesis from Arabidopsis thaliana. *Gene* 134 217–221. 10.1016/0378-1119(93)90096-L 8262380

[B46] Grierson D. (2014). “Ethylene Biosynthesis,” in *Fruit Ripening Physiology, Signalling and Genomics*, eds Nath P. Bouzayen M. Mattoo A. K. Pech J. C. (Oxfordshire: CAB International), 178–192.

[B47] Guis M. Bouquin T. Zegzouti H. Ayub R. Ben Amor M. Lasserre E. (1997). “Differential Expression of ACC Oxidase Genes in Melon and Physiological Characterization of Fruit Expressing an Antisense ACC Oxidase Gene,” in *Biology and Biotechnology of the Plant Hormone Ethylene*, eds Kanellis A. K. Chang C. Kende H. Grierson D. (Dordrecht: Springer), 327–337. 10.1007/978-94-011-5546-5_40

[B48] Guy M. Kende H. (1984). Conversion of 1-aminocyclopropane-1-carboxylic acid to ethylene by isolated vacuoles of Pisum sativum L. *Planta* 160 281–287. 10.1007/BF00402867 24258513

[B49] Hamilton A. J. Bouzayen M. Grierson D. (1991). Identification of a tomato gene for the ethylene-forming enzyme by expression in yeast. *Proc. Natl. Acad. Sci. U.S.A.* 88 7434–7437. 10.1073/pnas.88.16.7434 1714605 PMC52310

[B50] Hamilton A. J. Lycett G. W. Grierson D. (1990). Antisense gene that inhibits synthesis of the hormone ethylene in transgenic plants. *Nature* 346 284–287. 10.1038/346284a0

[B51] Han Y. Kuang J. Chen J. Liu X. Xiao Y. Fu C. (2016). Banana transcription factor MaERF11 recruits histone deacetylase MaHDA1 and represses the expression of MaACO1 and expansins during fruit ripening. *Plant Physiol.* 171:00301.2016. 10.1104/pp.16.00301 27208241 PMC4902611

[B52] Henzi M. X. McNeil D. L. Christey M. C. Lill R. E. (1999). A tomato antisense 1-aminocyclopropane-1-carboxylic acid oxidase gene causes reduced ethylene production in transgenic broccoli. *Funct. Plant Biol.* 26:179 10.1071/PP98083

[B53] Holdsworth M. J. Schuch W. Grierson D. (1987). Nucleotide sequence of an ethylene-related gene from tomato. *Nucleic Acids Res.* 15:10600. 10.1093/nar/15.24.10600 3697103 PMC339980

[B54] Huang L.-C. Lai U.-L. Yang S.-F. Chu M.-J. Kuo C.-I. Tsai M.-F. (2007). Delayed flower senescence of *Petunia hybrida* plants transformed with antisense broccoli ACC synthase and ACC oxidase genes. *Postharvest Biol. Technol.* 46 47–53. 10.1016/j.postharvbio.2007.03.015

[B55] Huang S. Sawaki T. Takahashi A. Mizuno S. Takezawa K. Matsumura A. (2010). Melon EIN3-like transcription factors (CmEIL1 and CmEIL2) are positive regulators of an ethylene- and ripening-induced 1-aminocyclopropane-1-carboxylic acid oxidase gene (CM-ACO1). *Plant Sci.* 178 251–257. 10.1016/j.plantsci.2010.01.005

[B56] Hudgins J. W. Ralph S. G. Franceschi V. R. Bohlmann J. (2006). Ethylene in induced conifer defense: cDNA cloning, protein expression, and cellular and subcellular localization of 1-aminocyclopropane-1-carboxylate oxidase in resin duct and phenolic parenchyma cells. *Planta* 224 865–877. 10.1007/s00425-006-0274-4 16705404

[B57] Iwai T. Miyasaka A. Seo S. Ohashi Y. (2006). Contribution of ethylene biosynthesis for resistance to blast fungus infection in young rice plants. *Plant Physiol.* 142 1202–1215. 10.1104/pp.106.085258 17012402 PMC1630725

[B58] Jafari Z. Haddad R. Hosseini R. Garoosi G. (2013). Cloning, identification and expression analysis of ACC oxidase gene involved in ethylene production pathway. *Mol. Biol. Rep.* 40 1341–1350. 10.1007/s11033-012-2178-7 23076530

[B59] Jia H. Chen S. Liu D. Liesche J. Shi C. Wang J. (2018). Ethylene-induced hydrogen sulfide negatively regulates ethylene biosynthesis by persulfidation of ACO in tomato under osmotic stress. *Front. Plant Sci.* 9:1517. 10.3389/fpls.2018.01517 30386366 PMC6199894

[B60] John I. Drake R. Farrell A. Cooper W. Lee P. Horton P. (1995). Delayed leaf senescence in ethylene-deficient ACC-oxidase antisense tomato plants: molecular and physiological analysis. *Plant J.* 7 483–490. 10.1046/j.1365-313X.1995.7030483.x

[B61] Kadyrzhanova D. McCully T. J. Warner T. Vlachonasios K. Wang Z. Dilley D. R. (1999). “Analysis of ACC Oxidase Activity by Site-Directed Mutagenesis of Conserved Amino Acid Residues,” in *Biology and Biotechnology of the Plant Hormone Ethylene II*, eds Kanellis A. K. (Dordrecht: Springer), 7–12. 10.1007/978-94-011-4453-7_2

[B62] Kawai Y. Ono E. Mizutani M. (2014). Evolution and diversity of the 2-oxoglutarate-dependent dioxygenase superfamily in plants. *Plant J.* 78 328–343. 10.1111/tpj.12479 24547750

[B63] Kende H. (1989). Enzymes of ethylene biosynthesis. *Plant Physiol.* 91 1–4. 10.1104/pp.91.1.1 16666977 PMC1061940

[B64] Klepikova A. V. Kasianov A. S. Gerasimov E. S. Logacheva M. D. Penin A. A. (2016). A high resolution map of the *Arabidopsis thaliana* developmental transcriptome based on RNA-seq profiling. *Plant J.* 88 1058–1070. 10.1111/tpj.13312 27549386

[B65] Knoester M. Linthorst H. J. M. Bol J. F. van Loon L. (1997). Modulation of stress-inducible ethylene biosynthesis by sense and antisense gene expression in tobacco. *Plant Sci.* 126 173–183. 10.1016/S0168-9452(97)00097-6

[B66] Kou X. Liu C. Han L. Wang S. Xue Z. (2016). NAC transcription factors play an important role in ethylene biosynthesis, reception and signaling of tomato fruit ripening. *Mol. Genet. Genom.* 291 1205–1217. 10.1007/s00438-016-1177-0 26852223

[B67] Li L. Wang X. Zhang X. Guo M. Liu T. (2017). Unraveling the target genes of RIN transcription factor during tomato fruit ripening and softening. *J. Sci. Food Agric.* 97 991–1000. 10.1002/jsfa.7825 27247090

[B68] Lieberman M. Kunishi A. Mapson L. W. Wardale D. A. (1966). Stimulation of ethylene production in apple tissue slices by methionine. *Plant Physiol.* 41 376–382. 10.1104/pp.41.3.376 16656267 PMC1086352

[B69] Lin Z. Hong Y. Yin M. Li C. Zhang K. Grierson D. (2008). A tomato HD-Zip homeobox protein. LeHB-1, plays an important role in floral organogenesis and ripening. *Plant J.* 55 301–310. 10.1111/j.1365-313X.2008.03505.x 18397374 PMC2607530

[B70] Lin Z. Zhong S. Grierson D. (2009). Recent advances in ethylene research. *J. Exp. Bot.* 60 3311–3336. 10.1093/jxb/erp204 19567479

[B71] Linkies A. Leubner-Metzger G. (2012). Beyond gibberellins and abscisic acid: how ethylene and jasmonates control seed germination. *Plant Cell Rep.* 31 253–270. 10.1007/s00299-011-1180-1 22044964

[B72] Linkies A. Muller K. Morris K. Tureckova V. Wenk M. Cadman C. S. C. (2009). Ethylene interacts with abscisic acid to regulate endosperm rupture during germination: a comparative approach using lepidium sativum and *Arabidopsis thaliana*. *Plant Cell* 21 3803–3822. 10.1105/tpc.109.070201 20023197 PMC2814513

[B73] Liu H. Yu H. Tang G. Huang T. (2018). Small but powerful: function of microRNAs in plant development. *Plant Cell Rep.* 37 515–528. 10.1007/s00299-017-2246-5 29318384

[B74] Liu J. Liu L. Li Y. Jia C. Zhang J. Miao H. (2015). Role for the banana AGAMOUS-like gene MaMADS7 in regulation of fruit ripening and quality. *Physiol. Plant.* 155 217–231. 10.1111/ppl.12348 25980771

[B75] Liu W. Meng J. Cui J. Luan Y. (2017). Characterization and function of MicroRNA^∗^s in Plants. *Front. Plant Sci.* 8:2200. 10.3389/fpls.2017.02200 29312425 PMC5744440

[B76] López-Gómez R. Cabrera-Ponce J. L. Saucedo-Arias L. J. Carreto-Montoya L. Villanueva-Arce R. Díaz-Perez J. C. (2009). Ripening in papaya fruit is altered by ACC oxidase cosuppression. *Transgenic Res.* 18 89–97. 10.1007/s11248-008-9197-0 18612838

[B77] Love J. Bjorklund S. Vahala J. Hertzberg M. Kangasjarvi J. Sundberg B. (2009). Ethylene is an endogenous stimulator of cell division in the cambial meristem of Populus. *Proc. Natl. Acad. Sci. U.S.A.* 106 5984–5989. 10.1073/pnas.0811660106 19293381 PMC2657089

[B78] Martel C. Vrebalov J. Tafelmeyer P. Giovannoni J. J. (2011). The tomato MADS-box transcription factor ripening inhibitor interacts with promoters involved in numerous ripening processes in a colorless nonripening-dependent manner. *Plant Physiol.* 157 1568–1579. 10.1104/pp.111.181107 21941001 PMC3252172

[B79] Martin M. N. Saftner R. A. (1995). Purification and characterization of 1-aminocyclopropane-1-carboxylic acid n-malonyltransferase from tomato fruit. *Plant Physiol.* 108 1241–1249. 10.1104/pp.108.3.1241 12228541 PMC157479

[B80] Martinez S. Hausinger R. P. (2015). Catalytic mechanisms of Fe(II)- and 2-Oxoglutarate-dependent oxygenases. *J. Biol. Chem.* 290 20702–20711. 10.1074/jbc.R115.648691 26152721 PMC4543632

[B81] Matarasso N. Schuster S. Avni A. (2005). A Novel Plant cysteine protease has a dual function as a regulator of 1-aminocyclopropane-1-carboxylic acid synthase gene expression. *Plant Cell* 17 1205–1216. 10.1105/tpc.105.030775 15749766 PMC1087997

[B82] Mayne R. G. Kende H. (1986). Ethylene biosynthesis in isolated vacuoles of Vicia faba L. - requirement for membrane integrity. *Planta* 167 159–165. 10.1007/BF00391410 24241846

[B83] Mitchell T. Porter A. J. R. John P. (1988). Authentic activity of the ethylene-forming enzyme observed in membranes obtained from kiwifruit (Actinidia deliciosa). *New Phytol.* 109 313–319. 10.1111/j.1469-8137.1988.tb04200.x

[B84] Müller M. Munné-Bosch S. (2015). Ethylene response factors: a key regulatory hub in hormone and stress signaling. *Plant Physiol.* 169 32–41. 10.1104/pp.15.00677 26103991 PMC4577411

[B85] Murphy L. J. Werner-zwanziger U. Moilanen J. Tuononen H. M. Clyburne J. A. C. (2014). A simple complex on the verge elusive cyanoformate ion. *Science* 75 75–79. 10.1126/science.1250808 24700853

[B86] Murr D. P. Yang S. F. (1975). Conversion of 5’-methylthioadenosine to methionine by apple tissue. *Phytochemistry* 14 1291–1292. 10.1016/S0031-9422(00)98613-8

[B87] Nakatsuka A. Murachi S. Okunishi H. Shiomi S. Nakano R. Kubo Y. (1998). Differential expression and internal feedback regulation of 1-aminocyclopropane-1-carboxylate synthase, 1-aminocyclopropane-1-carboxylate oxidase, and ethylene receptor genes in tomato fruit during development and ripening. *Plant Physiol.* 118 1295–1305. 10.1104/pp.118.4.1295 9847103 PMC34745

[B88] Nakabayashi K. Okamoto M. Koshiba T. Kamiya Y. Nambara E. (2005). Genome-wide profiling of stored mRNA in *Arabidopsis thaliana* seed germination: epigenetic and genetic regulation of transcription in seed. *Plant J.* 41 697–709. 10.1111/j.1365-313X.2005.02337.x 15703057

[B89] Nuñez-Palenius H. G. Cantliffe D. J. Huber D. J. Ciardi J. Klee H. J. (2006). Transformation of a muskmelon ‘Galia’ hybrid parental line (Cucumis melo L. var. reticulatus Ser.) with an antisense ACC oxidase gene. *Plant Cell Rep.* 25 198–205. 10.1007/s00299-005-0042-0 16362302

[B90] Ohme-Takagi M. Hideaki S. (1995). Ethylene-inducible DNA binding proteins that interact with an ethylene-responsive element. *Plant Cell* 7 173–182. 10.1105/tpc.7.2.173 7756828 PMC160773

[B91] Park C. H. Roh J. Youn J. Son S. Park J. H. Kim S. Y. (2018). *Arabidopsis* ACC oxidase 1 coordinated by multiple signals mediates ethylene biosynthesis and is involved in root development. *Mol. Cells* 41 923–932. 10.14348/molcells.2018.0092 30352493 PMC6199567

[B92] Peck S. C. Reinhardt D. Olson D. C. Boller T. Kende H. (1992). Localization of the ethylene-forming enzyme from tomatoes, 1-aminocyclopropane-1-carboxylate oxidase, in transgenic yeast. *J. Plant Physiol.* 140 681–686. 10.1016/S0176-1617(11)81023-0

[B93] Peiser G. D. Wang T.-T. Hoffman N. E. Yang S. F. Liu H. Walsh C. T. (1984). Formation of cyanide from carbon 1 of 1-aminocyclopropane-1-carboxylic acid during its conversion to ethylene. *Proc. Natl. Acad. Sci. U.S.A.* 81 3059–3063. 10.1073/pnas.81.10.3059 16593463 PMC345220

[B94] Picton S. Barton S. L. Bouzayen M. Hamilton A. J. Grierson D. (1993). Altered fruit ripening and leaf senescence in tomatoes expressing an antisense ethylene-forming enzyme transgene. *Plant J.* 3 469–481. 10.1111/j.1365-313X.1993.tb00167.x

[B95] Pommerrenig B. Feussner K. Zierer W. Rabinovych V. Klebl F. Feussner I. (2011). Phloem-specific expression of yang cycle genes and identification of novel yang cycle enzymes in plantago and *Arabidopsis*. *Plant Cell* 23 1904–1919. 10.1105/tpc.110.079657 21540433 PMC3123959

[B96] Porter A. J. R. Borlakoglu J. T. John P. (1986). Activity of the ethylene-forming enzyme in relation to plant cell structure and organization. *J. Plant Physiol.* 125 207–216. 10.1016/S0176-1617(86)80143-2

[B97] Proost S. Bel M. Van Vaneechoutte D. Van de Peer Y. Mueller-roeber B. Vandepoele K. (2014). PLAZA 3.0: an access point for plant comparative genomics. *Nucleic Acids Res.* 43 974–981. 10.1093/nar/gku986 25324309 PMC4384038

[B98] Ramassamy S. Olmos E. Bouzayen M. Pech J. Latché A. (1998). 1-aminocyclopropane-1-carboxylate oxidase of apple fruit is periplasmic. *J. Exp. Bot.* 49 1909–1915. 10.1093/jexbot/49.329.1909

[B99] Rauf M. Arif M. Fisahn J. Xue G.-P. Balazadeh S. Mueller-Roeber B. (2013). NAC transcription factor speedy hyponastic growth regulates flooding-induced leaf movement in *Arabidopsis*. *Plant Cell* 25 4941–4955. 10.1105/tpc.113.117861 24363315 PMC3903997

[B100] Raz V. Ecker J. R. (1999). Regulation of differential growth in the apical hook of Arabidopsis. *Development* 126 3661–3668. 10409511 10.1242/dev.126.16.3661

[B101] Reinhardt D. Kende H. Boiler T. (1994). Subcellular localization of 1-aminocyclopropane-1-carboxylate oxidase in tomato cells. *Planta* 195 142–146. 10.1007/BF00206302

[B102] Rombaldi C. Lelièvre J.-M. Latché A. Petitprez M. Bouzayen M. Pech J.-C. (1994). Immunocytolocalization of 1-aminocyclopropane-1-carboxylic acid oxidase in tomato and apple fruit. *Planta* 192 453–460. 10.1007/BF00203582 7764617

[B103] Savin K. W. Baudinette S. C. Graham M. W. Michael M. Z. Nugent G. D. Lu C.-Y. (1995). Antisense ACC Oxidase RNA delays carnation petal senescence. *HortScience* 30 970–972. 10.21273/HORTSCI.30.5.970

[B104] Schmid M. Davison T. S. Henz S. R. Pape U. J. Demar M. Vingron M. (2005). A gene expression map of *Arabidopsis thaliana* development. *Nat. Genet.* 37 501–506. 10.1038/ng1543 15806101

[B105] Sekeli R. Abdullah J. Namasivayam P. Muda P. Bakar U. Yeong W. (2014). RNA interference of 1-aminocyclopropane-1-carboxylic acid oxidase (aco1 and aco2) genes expression prolongs the shelf life of eksotika (*Carica papaya* L.) papaya fruit. *Molecules* 19 8350–8362. 10.3390/molecules19068350 24950439 PMC6270959

[B106] Sell S. Hehl R. (2005). A fifth member of the tomato 1-aminocyclopropane-1-carboxylic acid (ACC) oxidase gene family harbours a leucine zipper and is anaerobically induced. *J. DNA Seq. Mapp.* 16 80–82. 10.1080/10425170500050817 16040352

[B107] Seo Y. S. Yoo A. Jung J. Sung S.-K. Yang D. R. Kim W. T. (2004). The active site and substrate-binding mode of 1-aminocyclopropane-1-carboxylate oxidase determined by site-directed mutagenesis and comparative modelling studies. *Biochem. J.* 380 339–346. 10.1042/bj20031762 14972027 PMC1224174

[B108] Shaw J. Chou Y. Chang R. Yang S. F. (1996). Characterization of the ferrous ion binding sites of apple 1-aminocyclopropane-1-carboxylate oxidase by site-directed Mutagenesis. *Biochem. Biophys. Res. Commun.* 700 697–700. 10.1006/bbrc.1996.1237 8780676

[B109] Shi Y.-H. Zhu S.-W. Mao X.-Z. Feng J.-X. Qin Y.-M. Zhang L. (2006). Transcriptome profiling. molecular biological, and physiological studies reveal a major role for ethylene in cotton fiber cell elongation. *Plant Cell* 18 651–664. 10.1105/tpc.105.040303 16461577 PMC1383640

[B110] Silva J. A. Da Costa T. S. Lucchetta L. Marini L. J. Zanuzo M. R. Nora L. (2004). Characterization of ripening behavior in transgenic melons expressing an antisense 1-aminocyclopropane-1-carboxylate (ACC) oxidase gene from apple. *Postharvest Biol. Technol.* 32 263–268. 10.1016/j.postharvbio.2004.01.002

[B111] Staswick P. E. Tiryaki I. (2004). The oxylipin signal jasmonic acid is activated by an enzyme that conjugates it to isoleucine in *Arabidopsis*. *Plant Cell* 16 2117–2127. 10.1105/tpc.104.023549 15258265 PMC519202

[B112] Sun X. Li Y. He W. Ji C. Xia P. Wang Y. (2017). Pyrazinamide and derivatives block ethylene biosynthesis by inhibiting ACC oxidase. *Nat. Commun.* 8:15758. 10.1038/ncomms15758 28604689 PMC5472784

[B113] Tan Y. Liu J. Huang F. Guan J. Zhong S. Tang N. (2014). PhGRL2 protein, interacting with PhACO1, is involved in flower senescence in the petunia. *Mol. Plant* 7 1384–1387. 10.1093/mp/ssu024 24618881

[B114] Tierney D. L. Rocklin A. M. Lipscomb J. D. Que L. Hoffman B. M. (2005). ENDOR studies of the ligation and structure of the non-heme iron site in ACC oxidase. *J. Am. Chem. Soc.* 127 7005–7013. 10.1021/ja0500862 15884944

[B115] Tu Y. He B. Gao S. Guo D. Jia X. Dong X. (2019). CtACO1 overexpression resulted in the alteration of the flavonoids profile of safflower. *Molecules* 24:1128. 10.3390/molecules24061128 30901924 PMC6471848

[B116] Van de Poel B. Bulens I. Hertog M. L. A. T. M. Nicolai B. M. Geeraerd A. H. (2014a). A transcriptomics-based kinetic model for ethylene biosynthesis in tomato (Solanum lycopersicum) fruit: development, validation and exploration of novel regulatory mechanisms. *New Phytol.* 202 952–963. 10.1111/nph.12685 24443955

[B117] Van de Poel B. Vandenzavel N. Smet C. Nicolay T. Bulens I. Mellidou I. (2014b). Tissue specific analysis reveals a differential organization and regulation of both ethylene biosynthesis and E8 during climacteric ripening of tomato. *BMC Plant Biol.* 14:11. 10.1186/1471-2229-14-11 24401128 PMC3900696

[B118] Van de Poel B. Bulens I. Markoula A. Hertog M. L. A. T. M. Dreesen R. Wirtz M. (2012). Targeted systems biology profiling of tomato fruit reveals coordination of the Yang cycle and a distinct regulation of ethylene biosynthesis during postclimacteric ripening. *Plant Physiol.* 160 1498–1514. 10.1104/pp.112.206086 22977280 PMC3490579

[B119] Van de Poel B. Smet D. Van Der Straeten D. (2015). Ethylene and hormonal cross talk in vegetative growth and development. *Plant Physiol.* 169 61–72. 10.1104/pp.15.00724 26232489 PMC4577414

[B120] Van de Poel B. Van Der Straeten D. (2014). 1-aminocyclopropane-1-carboxylic acid (ACC) in plants: more than just the precursor of ethylene! *Front. Plant Sci.* 5:640. 10.3389/fpls.2014.00640 25426135 PMC4227472

[B121] van Es S. W. Silveira S. R. Rocha D. I. Bimbo A. Martinelli A. P. Dornelas M. C. (2018). Novel functions of the *Arabidopsis* transcription factor TCP5 in petal development and ethylene biosynthesis. *Plant J.* 94 867–879. 10.1111/tpj.13904 29570883 PMC6001666

[B122] Vandenbussche F. Vriezen W. H. Smalle J. Laarhoven L. J. J. Harren F. J. M. Van Der Straeten D. (2003). Ethylene and auxin control the *Arabidopsis* response to decreased light intensity. *Plant Physiol.* 133 517–527. 10.1104/pp.103.022665 12972669 PMC219028

[B123] Ververidis P. John P. (1991). Complete recovery in vitro of ethylene-forming activity. *Phytochemistry* 30 725–727. 10.1016/0031-9422(91)85241-q

[B124] Vriezen W. H. Hulzink R. Mariani C. Voesenek L. A. (1999). 1-aminocyclopropane-1-carboxylate oxidase activity limits ethylene biosynthesis in Rumex palustris during submergence. *Plant Physiol.* 121 189–196. 10.1104/pp.121.1.189 10482674 PMC59367

[B125] Waese J. Fan J. Pasha A. Yu H. Fucile G. Shi R. (2017). ePlant: visualizing and exploring multiple levels of data for hypothesis generation in plant biology. *Plant Cell* 29 1806–1821. 10.1105/tpc.17.00073 28808136 PMC5590499

[B126] Wang Y. Zou W. Xiao Y. Cheng L. Liu Y. Gao S. (2018). MicroRNA1917 targets CTR4 splice variants to regulate ethylene responses in tomato. *J. Exp. Bot.* 69 1011–1025. 10.1093/jxb/erx469 29365162

[B127] Wen C.-K. (ed.) (2015). *Ethylene in Plants.* Dordrecht: Springer 10.1007/978-94-017-9484-8

[B128] Xiong A.-S. Yao Q.-H. Peng R.-H. Li X. Han P.-L. Fan H.-Q. (2005). Different effects on ACC oxidase gene silencing triggered by RNA interference in transgenic tomato. *Plant Cell Rep.* 23 639–646. 10.1007/s00299-004-0887-7 15503033

[B129] Yoo A. Seo Y. S. Jung J. W. Sung S. K. Kim W. T. Lee W. (2006). Lys296 and Arg299 residues in the C-terminus of MD-ACO1 are essential for a 1-aminocyclopropane-1-carboxylate oxidase enzyme activity. *J. Struct. Biol.* 156 407–420. 10.1016/j.jsb.2006.08.012 17046279

[B130] Yoon G. M. (2015). New insights into the protein turnover regulation in ethylene biosynthesis. *Mol. Cells* 38 597–603. 10.14348/molcells.2015.0152 26095506 PMC4507024

[B131] Yu Y. Jia T. Chen X. (2017). The ‘how’ and ‘where’ of plant microRNAs. *New Phytol.* 216 1002–1017. 10.1111/nph.14834 29048752 PMC6040672

[B132] Zhang X. Wang W. Wang M. Zhang H.-Y. Liu J.-H. (2016). The miR396b of *Poncirus trifoliata* functions in cold tolerance by regulating acc oxidase gene expression and modulating ethylene–polyamine homeostasis. *Plant Cell Physiol.* 57 1865–1878. 10.1093/pcp/pcw108 27402968

[B133] Zhang X.-N. Li X. Liu J.-H. (2014). Identification of conserved and novel cold-responsive microRNAs in trifoliate orange (*Poncirus trifoliata* (L.) Raf.) Using High-Throughput Sequencing. *Plant Mol. Biol. Rep.* 32 328–341. 10.1007/s11105-013-0649-1

[B134] Zhang Z. Ren J.-S. Clifton I. J. Schofield C. J. (2004). Crystal structure and mechanistic implications of 1-aminocyclopropane-1-carboxylic acid oxidase—the ethylene-forming enzyme. *Chem. Biol.* 11 1383–1394. 10.1016/j.chembiol.2004.08.012 15489165

[B135] Zhang Z. Zhang H. Quan R. Wang X.-C. Huang R. (2009). Transcriptional regulation of the ethylene response factor LeERF2 in the expression of ethylene biosynthesis genes controls ethylene production in tomato and tobacco. *Plant Physiol.* 150 365–377. 10.1104/pp.109.135830 19261734 PMC2675746

