# Supplementary material for: 1-Aminocyclopropane-1-Carboxylic Acid Oxidase (ACO): The Enzyme That Makes the Plant Hormone Ethylene
Source: Front Plant Sci. 2019 May 29;10:695. doi: 10.3389/fpls.2019.00695 (PMC6549523; doi:10.3389/fpls.2019.00695)
Supplement: Supplementary file 1 [file Data_Sheet_1.docx]

Supplementary Material

Supplemental Table 1: List of all ACO sequences used in the phylogenetic analyses. BLASTp jobs were done for 21 species using the Phytozome (v12.1.) database and Gymno plaza 1.0 (Proost et al. 2014), and top hits were only retained after a positive reciprocal BLAST search. Numbering of the different ACOs was for each species done according to previously published work (if available), otherwise a new numbering is proposed. Putative splice variants of the same gene are represented by α, β, γ, δ and ε.

| Species | GeneID | Gene | Type | Protein (aa) | Source |
| --- | --- | --- | --- | --- | --- |
| *Amborella trichopoda* | AmTr_v1.0_scaffold00112.24 | *AmTrACO1* | 1 | 295 |  |
|  | AmTr_v1.0_scaffold00145.19 | *AmTrACO2* | 2 | 265 |  |
|  | AmTr_v1.0_scaffold00017.228 | *AmTrACO3* | 3 | 203 |  |
| Pineapple | Aco001358.1 | *AcoACO1* | 1 | 319 | (Cazzonelli et al. 1998) |
| *(Ananas comosus)* | Aco005735.1 | *AcoACO2* | 2 | 415 |  |
|  | Aco015240.1 | *AcoACO3* | 2 | 295 |  |
|  | Aco003285.1 | *AcoACO4* | 3 | 649 |  |
| *Arabidopsis thaliana* | AT2G19590.1 | *AtACO1* | 2 | 311 | (Vandenbussche et al. 2003) |
|  | AT1G62380.1 | *AtACO2* | 1 | 321 | (Raz and Ecker 1999) |
|  | AT1G12010.1 | *AtACO3* | 1 | 321 | (Vandenbussche et al. 2003) |
|  | AT1G05010.1 | *AtACO4* | 1 | 324 | (Gómez-Lim et al. 1993) |
|  | AT1G77330.1 | *AtACO5* | 3 | 308 | (Vandenbussche et al. 2003) |
| *Azolla filiculoides* | Azfi_s0076.g037826 |  |  | 396 |  |
|  | Azfi_s0335.g065519 |  |  | 388 |  |
| *Brachypodium distachyon* | Bradi3g57620.1 | *BdACO1* | 1 | 334 |  |
|  | Bradi4g31820.1 | *BdACO2* | 1 | 322 |  |
|  | Bradi1g37420.1 | *BdACO3* | 2 | 294 |  |
|  | Bradi2g41840.2 | *BdACO4* | 2 | 331 |  |
|  | Bradi2g35850.1 | *BdACO5* | 3 | 312 |  |
|  | Bradi2g35860.1 | *BdACO6* | 3 | 312 |  |
|  | Bradi4g23500.1 | *BdACO7* | 3 | 316 |  |
| *Chlamydomonas reinhardtii* | Cre10.g466700.t1.1 |  |  |  |  |
| Orange | orange1.1g026305m | *CiACO1* | 1 | 241 |  |
| (*Citrus sinensis*) | orange1.1g027317m | *CiACO2* | 1 | 226 |  |
|  | orange1.1g018920m | *CiACO3* | 1 | 350 |  |
|  | orange1.1g021002m | *CiACO4* | 1 | 319 |  |
|  | orange1.1g023985m | *CiACO5* | 1 | 275 |  |
|  | orange1.1g024020m | *CiACO6* | 1 | 275 |  |
|  | orange1.1g020953m | *CiACO7* | 1 | 320 |  |
|  | orange1.1g021636m | *CiACO8* | 2 | 311 |  |
|  | orange1.1g021590m | *CiACO9* | 3 | 311 |  |
| Cucumber | Cucsa.384740.1 | *CsACO1* | 1 | 315 | (Kahana et al. 1999) |
| (*Cucumis sativus*) | Cucsa.044880.1 | *CsACO2* | 1 | 318 | (Kahana et al. 1999) |
|  | Cucsa.185120.1 | *CsACO3* | 1 | 318 | (Kahana et al. 1999) |
|  | Cucsa.116950.1 | *CsACO4* | 2 | 301 |  |
|  | Cucsa.125280.1 | *CsACO5* | 3 | 310 |  |
| *Ginkgo biloba* | GBI00018194 |  |  | 345 |  |
| Soybean | Glyma.02G268000.1 | *GmACO1α* | 1 | 316 | (Arraes et al. 2015) |
| (*Glycine max*) | Glyma.02G268000.2 | *GmACO1β* | 1 | 203 |  |
|  | Glyma.02G268000.3 | *GmACO1γ* | 1 | 203 |  |
|  | Glyma.02G268000.4 | *GmACO1δ* | 1 | 256 |  |
|  | Glyma.02G268000.5 | *GmACO1ε* | 1 | 228 |  |
|  | Glyma.02G268200.1 | *GmACO2α* | 1 | 308 | (Arraes et al. 2015) |
|  | Glyma.02G268400.1 | *GmACO2β* | 1 | 198 |  |
|  | Glyma.07G264200.1 | *GmACO8* | 1 | 319 | (Arraes et al. 2015) |
|  | Glyma.08G050400.1 | *GmACO10* | 1 | 311 | (Arraes et al. 2015) |
|  | Glyma.09G008400.1 | *GmACO11* | 1 | 319 | (Arraes et al. 2015) |
|  | Glyma.14G048900.1 | *GmACO12* | 1 | 308 | (Arraes et al. 2015) |
|  | Glyma.14G049000.1 | *GmACO16α* | 1 | 308 |  |
|  | Glyma.14G049000.2 | *GmACO16β* | 1 | 308 |  |
|  | Glyma.14G049200.1 | *GmACO13* | 1 | 308 | (Arraes et al. 2015) |
|  | Glyma.14G049500.1 | *GmACO14α* | 1 | 316 | (Arraes et al. 2015) |
|  | Glyma.14G049500.2 | *GmACO14β* | 1 | 233 |  |
|  | Glyma.15G112700.1 | *GmACO15* | 1 | 319 | (Arraes et al. 2015) |
|  | Glyma.17G009800.1 | *GmACO17* | 1 | 320 |  |
|  | Glyma.05G222400.1 | *GmACO5* | 2 | 308 | (Arraes et al. 2015) |
|  | Glyma.07G129000.1 | *GmACO7α* | 2 | 307 | (Arraes et al. 2015) |
|  | Glyma.07G129000.2 | *GmACO7β* | 2 | 307 |  |
|  | Glyma.08G029200.1 | *GmACO9* | 2 | 308 | (Arraes et al. 2015) |
|  | Glyma.04G245900.1 | *GmACO4* | 3 | 309 | (Arraes et al. 2015) |
|  | Glyma.06G117200.1 | *GmACO6* | 3 | 308 | (Arraes et al. 2015) |
| Cotton | Gorai.009G182300.1 | *GrACO1α* | 1 | 320 | (Shi et al. 2006)* |
| *Gossypium raimondii* | Gorai.009G182300.2 | *GrACO1β* | 1 | 239 | (Shi et al. 2006)* |
|  | Gorai.010G185000.1 | *GrACO2α* | 1 | 289 | (Shi et al. 2006)* |
|  | Gorai.010G185000.2 | *GrACO2β* | 1 | 204 | (Shi et al. 2006)* |
|  | Gorai.001G217400.1 | *GrACO3* | 1 | 312 | (Shi et al. 2006)* |
|  | Gorai.001G096400.1 | *GrACO4* | 1 | 314 | (Shi et al. 2006)* |
|  | Gorai.004G062100.1 | *GrACO5α* | 1 | 318 |  |
|  | Gorai.004G062100.3 | *GrACO5β* | 1 | 205 |  |
|  | Gorai.004G062100.4 | *GrACO5γ* | 1 | 282 |  |
|  | Gorai.004G062100.5 | *GrACO5δ* | 1 | 240 |  |
|  | Gorai.007G170100.1 | *GrACO6* | 1 | 311 |  |
|  | Gorai.010G184900.1 | *GrACO7* | 1 | 329 |  |
|  | Gorai.010G185100.1 | *GrACO8* | 1 | 210 |  |
|  | Gorai.001G011100.1 | *GrACO9* | 2 | 305 |  |
|  | Gorai.005G087900.1 | *GrACO10* | 3 | 323 |  |
|  | Gorai.005G088000.1 | *GrACO11* | 3 | 308 |  |
|  | Gorai.013G107500.1 | *GrACO12* | 3 | 304 |  |
| Apple | MDP0000195885 | *MdACO1* | 1 | 314 | (Dong et al. 1992) |
| (*Malus domestica*) | MDP0000200737 | *MdACO2* | 1 | 330 | (Binnie and McManus 2009) |
|  | MDP0000725984 | *MdACO3* | 1 | 323 | (Binnie and McManus 2009) |
|  | MDP0000251295 | *MdACO4* | 1 | 322 |  |
|  | MDP0000453114 | *MdACO5* | 1 | 323 |  |
|  | MDP0000025650 | *MdACO6* | 3 | 298 |  |
|  | MDP0000200896 | *MdACO7* | 2 | 348 |  |
| *Marchantia polymorpha* | Mapoly0019s0012.1 |  |  | 396 |  |
|  | Mapoly0067s0061.1 |  |  | 346 |  |
| Rice | LOC_Os09g27820.1 | *OsACO1* | 1 | 323 | (Chae et al. 2000) |
| (*Oryza sativa*) | LOC_Os09g27750.1 | *OsACO2* | 1 | 323 | (Chae et al. 2000) |
|  | LOC_Os02g53180.1 | *OsACO3α* | 1 | 345 | (Chae et al. 2000) |
|  | LOC_Os02g53180.2 | *OsACO3β* | 1 | 322 | (Chae et al. 2000) |
|  | LOC_Os02g53180.3 | *OsACO3γ* | 1 | 284 | (Chae et al. 2000) |
|  | LOC_Os06g37590.1 | *OsACO6* | 2 | 294 |  |
|  | LOC_Os01g39860.1 | *OsACO7* | 2 | 313 | (Iwai et al. 2006) |
|  | LOC_Os11g08380.1 | *OsACO4* | 3 | 310 | (Iwai et al. 2006) |
|  | LOC_Os05g05680.1 | *OsACO5* | 3 | 309 | (Iwai et al. 2006) |
| *Penium exiguum* | lcl\|Peex-YSQT-2032431 |  |  | 155 |  |
|  | lcl\|Peex-YSQT-2036798 |  |  | 322 |  |
| *Physcomitrella patens* | Pp3c4_3409V3.1 |  |  | 343 |  |
|  | Pp3c12_18540V3.1 |  |  | 354 |  |
|  | Pp3c12_18540V3.2 |  |  | 354 |  |
| *Pinus taeda* | PTA00000890 | *PtACO1* | 3 | 333 | (Yuan et al. 2010) |
|  | PTA00083080 | *PtACO2* | 3 | 336 | (Yuan et al. 2010) |
|  | PTA00031159 | *PtACO3* | 3 | 324 | (Yuan et al. 2010) |
|  | PTA00000141 | *PtACO4* | 3 | 233 |  |
| *Salvinia cucullata* | Sacu_v1.1_s0038.g011978 |  |  | 404 |  |
|  | Sacu_v1.1_s0057.g014747 |  |  | 358 |  |
|  | Sacu_v1.1_s0089.g018698 |  |  | 348 |  |
| *Selaginella moellendorffii* | Sm79989 |  |  | 357 |  |
|  | Sm85121 |  |  | 344 |  |
|  | Sm92217 |  |  | 348 |  |
|  | Sm167760 |  |  | 346 |  |
|  | Sm167765 |  |  | 346 |  |
|  | Sm439368 |  |  | 346 |  |
| Tomato | Solyc07g049530.2.1 | *SlACO1* | 1 | 316 | (Hamilton et al. 1991) |
| (*Solanum lycopersicum*) | Solyc12g005940.1.1 | *SlACO2* | 1 | 317 | (Holdsworth et al. 1987) |
|  | Solyc07g049550.2.1 | *SlACO3* | 1 | 317 | (Bidonde et al. 1998) |
|  | Solyc02g081190.2.1 | *SlACO4* | 1 | 321 | (Nakatsuka et al. 1998) |
|  | Solyc07g026650.2.1 | *SlACO5* | 2 | 302 | (Sell and Hehl 2005) |
|  | Solyc02g036350.2.1 | *SlACO6* | 1 | 320 |  |
|  | Solyc06g060070.2.1 | *SlACO7* | 3 | 315 |  |
| Maize | Zm00008a017510_T01 | *ZmACO20* | 1 | 453 | (Gallie and Young 2004) |
| (*Zea mays*) | Zm00008a023130_T01 | *ZmACO35* | 1 | 304 | (Gallie and Young 2004) |
|  | Zm00008a028217_T01 | *ZmACO2* | 1 | 327 |  |
|  | Zm00008a009058_T01 | *ZmACO8* | 2 | 239 |  |
|  | Zm00008a021339_T01 | *ZmACO9* | 2 | 235 |  |
|  | Zm00008a024831_T01 | *ZmACO1* | 2 | 283 |  |
|  | Zm00008a031986_T01 | *ZmACO10* | 2 | 319 |  |
|  | Zm00008a018191_T01 | *ZmACO6* | 3 | 314 |  |
|  | Zm00008a037498_T01 | *ZmACO31* | 3 | 315 | (Gallie and Young 2004) |
|  | Zm00008a037500_T01 | *ZmACO4* | 3 | 316 |  |
|  | Zm00008a037501_T01 | *ZmACO7* | 3 | 296 |  |
|  | Zm00008a037502_T01 | *ZmACO15* | 3 | 315 | (Gallie and Young 2004) |

*The publication of Shi et al. uses the species *Gossypium* *hirsutum* instead of *Gossypium* *raimondii*.

Supplemental Table 2: List of the important ACO residues studied in apple, tomato and Arabidopsis, and their confirmed/suggested function.

| Species | Gene | Residues | Function | Source |
| --- | --- | --- | --- | --- |
| Apple (*Malus domestica*) | *MdACO1* | H177, D179, H234 | Fe(II) binding site | (Shaw et al. 1996) |
| Apple  (*Malus domestica*) | *MdACO1* | H177, D179, H234 | Fe(II) binding site | (Kadyrzhanova D.K. et al. 1997) |
|  |  | C28 | Hypothesis: Essential for ACO function. Possible important role for the SH-group. |  |
|  |  | C133 | A putative leucine zipper |  |
|  |  | C165 | Hypothesis: important for protein-protein interaction, not for ACO enzyme function |  |
| Apple (*Malus domestica*) | *MdACO1* | H177, D179, H234 | Fe(II) binding site | (Kadyrzhanova et al. 1999) |
|  |  | K292, E297, R299, E301 | Important for CO_2_ activated enzyme function. |  |
|  |  | R244; S246 | ACC binding; ascorbate binding; activation by CO_2_ |  |
| *Petunia hybrida* | *ACO^a^* | H177, D179, H234 | Fe(II) binding site | (Zhang et al. 2004) |
|  |  | R175, R244 | Bicarbonate binding; hypothesis: involved in binding the complete Fe-ACC-O_2_- HCO_3_^-^-complex |  |
|  |  | R244, S246 | Part of RXS motif, binding of the carboxylate group of ACC and/or bicarbonate |  |
| Apple  (*Malus domestica*) | *MdACO1* | R244, S246 | Part of RXS motif, binding of ascorbate through hydrogen bonds | (Seo et al. 2004) |
|  |  |  |  |  |
|  |  |  |  |  |
| Apple  (*Malus domestica*) | *MdACO1* | K296, R299 | Important for enzyme function in the presence of ACC and the co-substrates | (Yoo et al. 2006) |
|  |  | H177, D179, H234 | Fe(II) binding site |  |
|  |  |  |  |  |
| Tomato | *SlACO1* | H177, D179 | Fe(II) binding site | (Brisson et al. 2012) |
| (*Solanum lycopersicum*) |  | K158, R300, S246, R244, Y162 | Involved in the interaction with HCO_3_^—^ACC |  |
|  |  | R300, R175, R235, V236 | Hypothesis: Interaction with ascorbate |  |
| Apple  (*Malus domestica*) | *MdACO^b^* | K158, R299, C-terminal α-helix 11 | Binding sites for ascorbate and bicarbonate to activate ACO | (Dilley et al. 2013) |
|  |  | R175, R244, S246, K158, K292, R299, F300 | Binding sites for ACC, bicarbonate and ascorbic acid; R244, S246 (in RXS motif) |  |
|  |  | E297, F300, E301 in α-helix 11 | Important for ACO reaction |  |
|  |  | T157 | Important for ACO activity; probably through stabilization of the β-strand containing K158 |  |
|  |  | C28, C133, C165 | Important for ACO enzyme function. |  |
|  |  | C28, T157, K158, R175, Q188, K199, K230, R244, S246, K292, E294, E297, R299, F300, E301 | Most important residues affecting ACO activity |  |
| *Arabidopsis thaliana* | *AtACO2^c^* | C63 | Target for *S*-glutathionylation | (Datta et al. 2015) |
| *Arabidopsis thaliana* | *AtACO2* | K161 | Binding of PA/POA (Instead of bicarbonate) | (Sun et al. 2017) |
|  |  | K291 | Binding of PA/POA |  |
|  |  | I187, L189, A251, F253 | Binding of PA/POA |  |
| Tomato  (*Solanum lycopersicum*) | *SlACO1* & *SlACO2* | C60 | S-sulfhydration target | (Jia et al. 2018) |

(a) It is unclear which *Petunia* *hybrida* ACO was studied. Based on the sequences published by Tang et al., the most likely candidate is ACO1 (Tang et al. 1993).

(b) In the publication of Dilley et al. (2013), E294 was indicated to be important for enzyme function. However, *Md*ACO1 (MDP0000195885) contains a glutamine at position 294 instead of a glutamic acid residue. E294 is retrieved in either *Md*ACO3 (MDP0000725984) or *Md*ACO5 (MDP0000453114).

(c) Datta et al. (2015) presented the S-glutathionylation of C63 in *At*ACO1. However, *At*ACO1 (AT2G19590.1) does not contain a cysteine residue in that position. Based on the sequence presented in the the study of Datta et al (2015), the actual ACO is *At*ACO2 (AT1G62380.1).


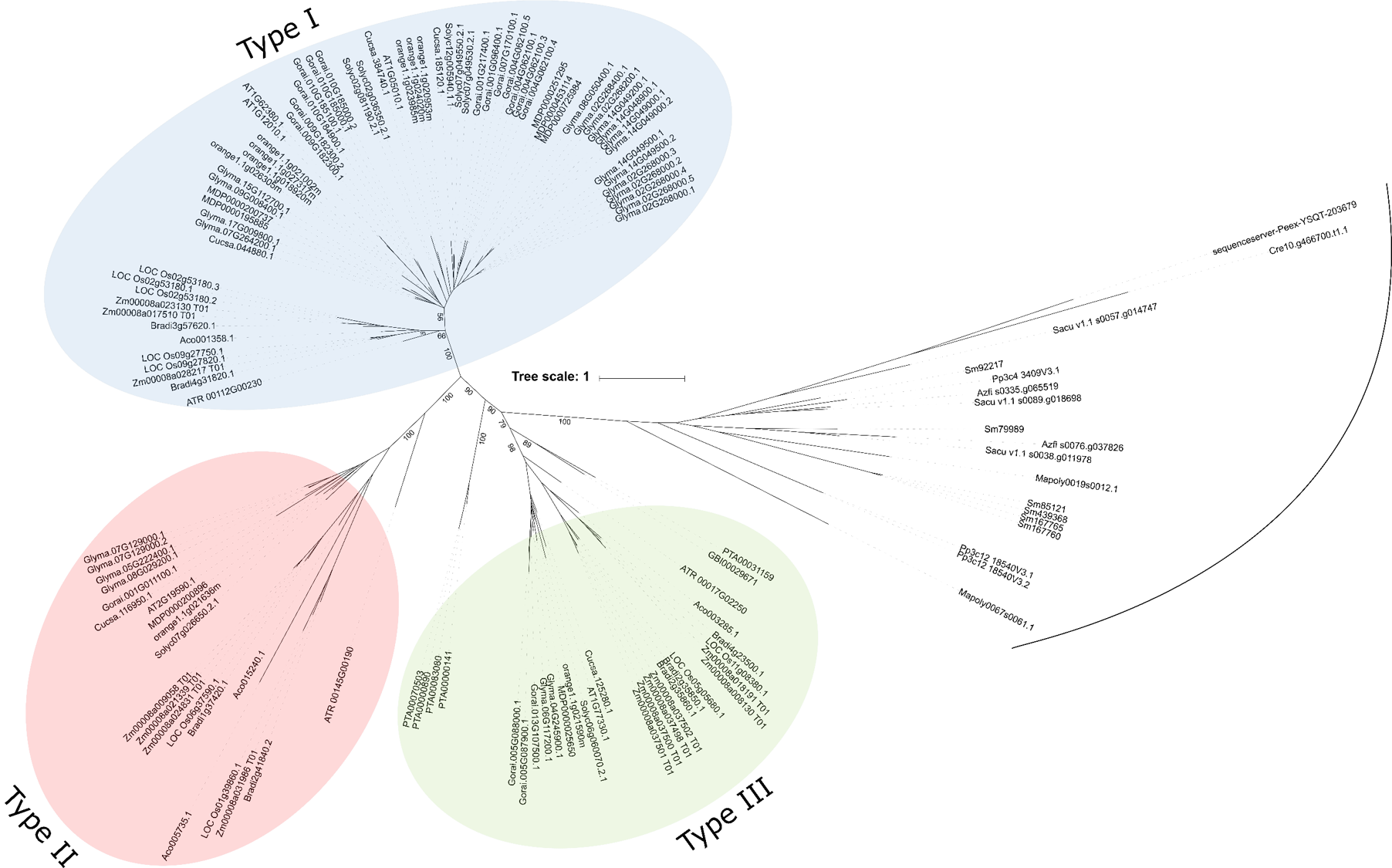


Supplemental Figure 1: Maximal likelihood phylogenetic tree for ACO protein sequences of *Amborella trichopoda* (ATR), Pineapple (*Ananas comosus*; Aco), *Arabidopsis thaliana* (AT), *Azolla filiculoides* (Azfi), *Brachypodium distachyon* (Bradi), *Chlamydomonas reinhardtii* (Cre), Orange (*Citrus sinensis*; orange), Cucumber (*Cucumis sativus*; Cucsa), *Ginkgo biloba* (GBI), Soybean (*Glycine max*; Glyma), Cotton (*Gossypium raimondii*; Gorai), Apple (*Malus domestica*; MDP), *Marchantia polymorpha* (Mapoly), Rice (*Oryza sativa*; Os), *Penium exiguum* (Peex), *Physcomitrella patens* (Pp), *Pinus taeda* (PTA), *Salvinia cucullata* (Sacu), *Selaginella moellendorffii* (Sm), Tomato (*Solanum lycopersicum*; Solyc) and Maize (*Zea mays*; Zm) retrieved from Phytozome (v12.1.) and Gymno plaza. Protein sequences were aligned in Geneious (v10.2.2) using the MUSCLE alignment plugin. The phylogenetic tree was build using RAxML (v8.2.11) for best-scoring maximum likelihood tree with rapid bootstrapping (1000 bootstrap replicates). Bootstrap values for the main branches are depicted on the tree. Type I ACO is shown in blue, Type II ACO is shown in red and Type III ACO is shown in green. Ancestral non-seed plant node of putative ACOs are also grouped with a brace.
